# Robust agricultural pest detection under occlusion and environmental variations via AGIIN-MAF training and SAODL adaptation

**DOI:** 10.3389/fpls.2026.1808419

**Published:** 2026-05-19

**Authors:** Changying Fan, Junbo Zhang, Shiyu Wang, Yantong Guo, Rui Fu

**Affiliations:** 1 Department of Information Science and Engineering, Weifang University of Science and Technology, Shouguang, Shandong, China; 2 School of Information and Artificial Intelligence, Shandong First Medical University, Tai’an, Shandong, China; 3 Department of Computer Engineering, Dongseo University, Busan, Republic of Korea

**Keywords:** convolutional autoencoder, deep learning, pest detection, test-time adaptation, uncertainty estimation

## Abstract

This study addresses the challenge of pest detection in agriculture, particularly focusing on improving the accuracy and robustness of detection models in varying environmental conditions and when pests are occluded. We propose a novel method, AGIIN-MAF, which includes a main and an auxiliary model. The auxiliary model, equipped with a convolutional autoencoder (CAE) and an Adaptive Gate Information Integration Network (AGIIN), processes occluded images to enhance the main model’s ability to detect pests even when they are partially hidden. Our method achieves an mAP50 of 80.2% in detecting occluded objects without increasing the number of model parameters. Furthermore, we introduce a Selective Adaptive Optimization for Domain-aware Learning (SAODL) strategy during the testing phase, which adapts the model to new, changing environments by distinguishing between domain-invariant and domainsensitive parameters. This approach yields a superior average mAP50 of 74.4% across various environmental conditions, outperforming other test-time adaptation methods. Our contributions include enhancing the model’s capability to detect occluded pests and improving its adaptability to unseen environments, which is crucial for effective pest management in agriculture.

## 1 Introduction

With the continuous change in climate, pest control has become a global challenge (Secretariat, 2021). Since 1970, the average rate of pest occurrence in China has increased by more than four times (Wang et al., 2021a). Pests significantly threaten crop yields and global food security, underscoring the growing urgency for effective pest control strategies. This is followed by the need for precise detection of pests to efficiently target specific pest species and their distribution locations.

**Figure 1:**
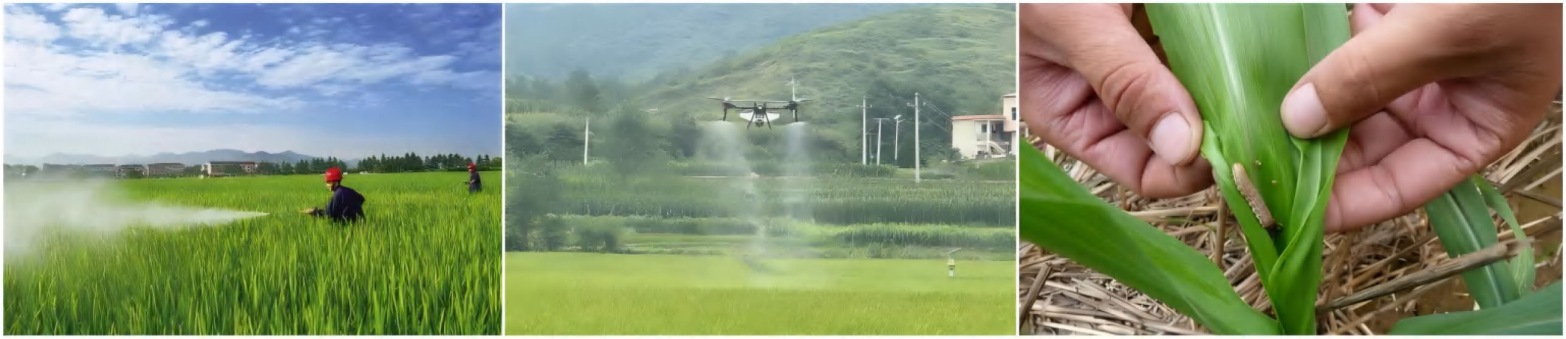
Traditional manual inspection of crop pests and large-scale extermination.

Traditional pest identification relies heavily on manual on-site observations (**Figure 1**), which are laborintensive and prone to misdiagnosis. To automate this process, various methods utilizing technologies such as radar imaging (Dedic et al., 2024), bio-inspired classifiers (Deng et al., 2018), and hyperspectral imaging (Nguyen et al., 2024) have been proposed. However, these existing approaches often demonstrate limited robustness, particularly when pests are partially occluded or subjected to complex environmental variations in real-world agricultural settings.

In addition, machine learning and computer vision approaches for image-based pest detection have gained prominence. Amrani et al. (2024) designed a Bayesian-based multi-task learning model and evaluated it on an agricultural pest dataset spanning corn, rapeseed, rice, and wheat, achieving aphid detection rates of 75.77%, 66.39%, 70.01%, and 59%, respectively. Kasinathan et al. (2021) developed an insect detection algorithm that attained the highest classification accuracies of 91.5% and 90% for nine- and twenty-four-class insect categories. Qiang et al. (2023) enhanced a single-shot multi-box detector model with a dual-backbone network, demonstrating robustness on the citrus pest dataset with an mAP of 86.01%. Saadati et al. (2024) investigated out-of-distribution detection algorithms to improve the robustness and reliability of insect classification models in practical scenarios. Ye et al. (2023) introduced a hybrid optimization strategy combining genetic algorithms with stochastic gradient descent to boost both accuracy and robustness in small pest detection under field conditions. Siva Sangari et al. (2016) employed ant colony optimization to refine fuzzy C-means clustering parameters, enhancing pest image segmentation performance. Collectively, these methods have substantially improved detection efficiency and accuracy while reducing dependence on specialized expertise, thereby accelerating pest monitoring and enabling earlier intervention.

Beyond agricultural applications, advanced computer vision and deep learning methodologies have driven remarkable progress across diverse and challenging domains. For instance, improved semantic segmentation algorithms have significantly enhanced the parsing of complex underwater scenes (Liu and Fang, 2020). In the medical field, foundation models leveraging pseudo-labels have substantially improved feature discrimination for 3D medical image segmentation (Jin et al., 2026). Furthermore, novel robust learning architectures, such as progressive sample selection frameworks with contrastive loss, have demonstrated exceptional capability in mitigating the adverse effects of noisy labels in large-scale datasets (Zhang et al., 2025). These cross-domain breakthroughs in robust feature extraction, uncertainty management, and complex environment adaptation provide profound methodological inspiration for addressing the unique occlusion and illumination challenges inherent in agricultural pest detection. Deep learning, a pivotal branch of machine learning, has further advanced pest detection by demonstrating superior feature representation and classification capabilities. Suthakaran and Premaratne (2020) developed a convolutional neural network (CNN) achieving 96.2% accuracy, 97.5% recall, and an F1 score of 0.982, significantly improving pest identification efficiency. Fang et al. (2024) introduced Pest-ConFormer, a hybrid CNN-Transformer model, attaining 77.81% accuracy on the IP102 dataset. Peng et al. (2024) designed the lightweight DC-GhostNet, which achieved 89.15% accuracy, 87.13% precision, 85.77% recall, and 86.45% F1 score on a 15-species rice pest dataset, while reducing model parameters and computational cost by 16% and 9%, respectively. Li et al. (2024) applied deep transfer learning to accurately identify pests and diseases in Yunnan large-leaf tea under complex environments, achieving test and validation accuracies of 98.58% and 98.23%, respectively. Sun et al. (2024) developed a real-time detection model with an mAP50 of 96.6%, surpassing baseline and YOLOv9s models by 4.1% and 2.0%, respectively. Ye et al. (2024) proposed PestNAS, which leverages adaptive feature fusion and evolutionary neural architecture search, enhancing detection accuracy for 11 pest types in field conditions. Other notable contributions include DMSAU-Net (Wang et al., 2024), TinySegformer (Zhang and Lv, 2024), AM-ResNet (Zhang et al., 2022), and MFFNet (Wei et al., 2022), which collectively improve performance, computational efficiency, and energy consumption in agricultural pest detection tasks. Similarly, AF-RCNN (Jiao et al., 2020) and deep residual learning-based approaches (Cheng et al., 2017) demonstrate robust multi-category pest identification with high classification accuracies in complex backgrounds.

The YOLO series has recently driven substantial progress in pest detection. Zhao et al. (2024a) proposed an improved YOLOv7 algorithm, achieving an AP of 91.9% and an F1 score of 89.6%, surpassing the original algorithm by 6.2 and 3.4 percentage points, respectively. Zhu et al. (2024) developed CBF-YOLO for precise detection of soybean pests under complex conditions, reaching an mAP of 86.9%. MD-YOLO (Tian et al., 2023) integrated DenseNet blocks with adaptive attention modules to enhance small pest detection, attaining mAP@0.5 of 86.2%, an F1 score of 79.1%, and IoU of 88.1%. Li et al. (2023) optimized YOLOv5 for passion fruit pest recognition, achieving 96.51% average precision and an average detection time of 7.7 ms.

Despite these advances, deep learning-based pest detection methods typically assume that data are independent and identically distributed (i.i.d.), limiting their robustness under distribution shifts and environmental noise. Test-Time Adaptation (TTA) has emerged as a promising solution, allowing models to adapt to target distributions without requiring labeled data by fine-tuning during testing. In pest detection, TTA improves both accuracy and adaptability to varying environmental conditions. For example, Ma (2024) proposed an improved self-training TTA approach that enhances pseudo-label quality and stabilizes optimization. Sójka et al. (2023) developed AR-TTA, achieving state-of-the-art performance through dynamic batch normalization updates combined with memory buffers and augmented data. Lee et al. (2023), Shin et al. (2022), and Cui et al. (2023) further demonstrated the effectiveness of TTA frameworks in pose estimation, 3D semantic segmentation, and human pose prediction, respectively.

A key challenge in TTA is catastrophic forgetting, whereby adaptation to new tasks leads to deterioration of previously learned knowledge. To mitigate this, uncertainty estimation has become a critical research focus. Zhou et al. (2021) highlighted the role of uncertainty quantification in enhancing interpretability in deep learning, particularly in medical imaging. Begoli et al. (2019) emphasized the need for formal uncertainty quantification (UQ) frameworks to bolster confidence in machine-assisted decision-making. Shamsi et al. (2021), Cheng et al. (2023), and Xue et al. (2019) further demonstrated the integration of uncertainty estimation with deep learning and Bayesian approaches, improving reliability across biomedical and dynamic system applications.

This study presents a method divided into two phases: training and testing. In the training phase, we introduce the AGIIN-MAF approach, comprising a main model and an auxiliary model, to enhance the model’s accuracy in detecting occluded objects and its robustness across varying environmental conditions. The main model remains unchanged and receives the original image input, while the auxiliary model receives randomly occluded image input. In the auxiliary model, we incorporated two convolutional autoencoders to enhance the model’s ability to detect damaged images and occluded objects. During training, after the first feature extraction layer C3K2, the main and auxiliary models dynamically exchange feature information through the Adaptive Gate Information Integration Network (AGIIN). The role of AGIIN is to promote the exchange of feature information between the main and auxiliary models in a beneficial direction, allowing the transmission of useful information while preventing the exchange of redundant or incorrect information, enabling both models to learn useful knowledge in this process. Subsequently, the auxiliary model performs image restoration through the Convolutional Autoencoder (CAE). After the first feature extraction layer C3K2 following the CAE in the auxiliary model, the main and auxiliary models exchange feature information again. This process is repeated twice throughout the entire training process. The main model acquires the capability to detect occluded objects and damaged images by exchanging feature information before and after the CAE in the auxiliary model. To improve the model’s detection accuracy while ensuring detection efficiency, we only retain the weights of the main model for detection after training is completed. Experiments show that our training method achieves an mAP50 of 80.2% in detecting occluded objects without increasing the model’s parameter quantity and complexity.

During the testing stage, to enable the model to adapt to constantly changing and distributionally inconsistent environments, inspired by the latest advancements in test-time adaptation and uncertainty estimation, we proposed an innovative domain adaptability analysis method. This method quantifies the sensitivity of model parameters to new target domains by distinguishing model parameters into domaininvariant and domain-sensitive parameters, and adopts a data-driven selective adaptation optimization strategy to assign different optimization strategies to these two types of parameters. In addition,the Selective Adaptive Optimization for Domain-aware Learning (SAODL) strategy separately updates domain-sensitive and domain-invariant parameters, thereby enhancing its adaptability to new environments while protecting the model’s generalization ability, achieving the highest mAP50 of 74.4% in constantly changing crossdomain environments, which is superior to other TTA methods. In summary, this work aims to improve the model’s detection accuracy and robustness in detecting occluded pests and unseen environments, with the following contributions:

- The AGIIN-MAF method, which incorporates Convolutional Autoencoder (CAE) and Adaptive Gate Information Integration Network (AGIIN), is proposed. It enhances the model’s ability to detect occluded objects and reconstruct or inpaint occluded regions through collaborative training of the main and auxiliary models, without increasing additional model parameter quantity and thereby improving the model’s robustness.
- Through two rounds of feature information exchange, the main model can learn and absorb the image restoration capability of the auxiliary model, thus acquiring the ability to detect partially occluded objects without relying on CAE. Meanwhile, a new domain adaptability analysis method is proposed to effectively evaluate the sensitivity of model parameters to new target domains, providing a scientific basis for the dynamic adjustment of the model.
- The Fisher Information Matrix (FIM) is introduced to identify domain-sensitive and domain-invariant parameters, achieving precise separation of parameters. This separation is governed by an empirical thresholding strategy designed to balance knowledge preservation and domain adaptation, laying the foundation for subsequent optimization strategies, such as the SAODL strategy.
- The SAODL strategy is designed to maintain the model’s memory of knowledge in the source domain while enhancing its adaptability to new domains, ensuring that the model can better handle distribution shifts across target domains and achieve a superior stability-plasticity balance.

## 2 Materials and methods

### 2.1 Training phase

**Figure 2:**
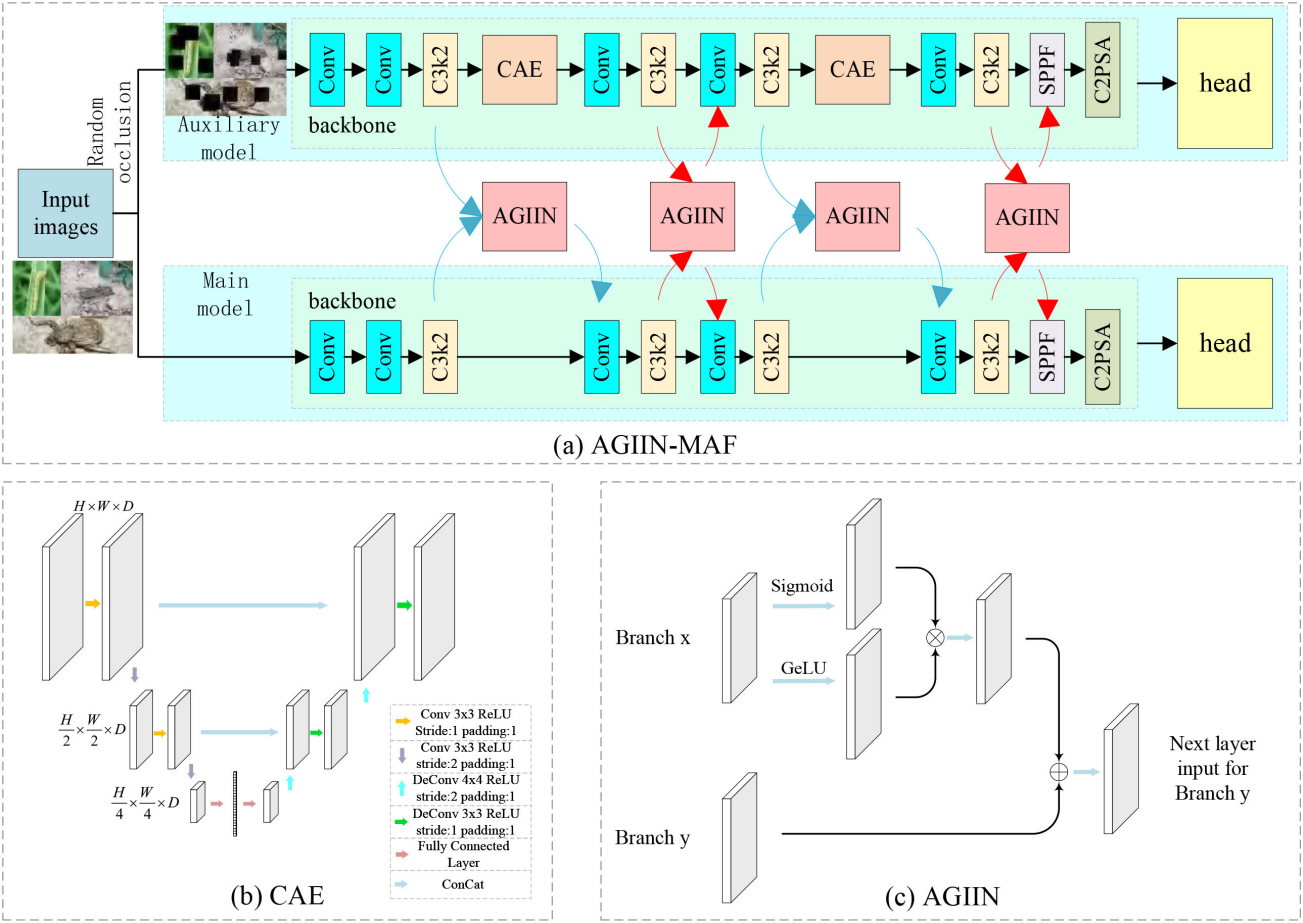
Overall training framework of the proposed AGIIN-MAF method. **(a)** During training, the main model processes the original clean image, while the auxiliary model receives the same image with random occlusion. The two branches share an identical backbone and neck architecture. Feature information is exchanged twice via the Adaptive Gate Information Integration Network (AGIIN, shown in **(c)**): as indicated by the blue arrows,the first exchange (after the 1st and 3rd C3K2 layers) is unidirectional (aux → main) to prevent the main model from seeing restored features too early;as indicated by the red arrows,the second exchange (after the 2nd and 4th C3K2 layers) is bidirectional. The auxiliary branch contains two Convolutional Autoencoders (CAE, detailed in **(b)**) that reconstruct occluded regions after the 1st and 3rd C3K2 layers, respectively. After training, only the main model is retained for inference.

In real application scenarios, pest detection is often affected by environmental changes and occlusions, leading to a decrease in the accuracy of detection results. To overcome this challenge, we propose the Adaptive Gate Information Integration Network-assisted Main-auxiliary Model Framework (AGIIN-MAF), as shown in **Figure 2a**. In this framework, the main model and the auxiliary model share the same structure, but they receive different types of inputs: the main model processes the original images, while the auxiliary model processes images that have been randomly occluded. To enhance the model’s ability to detect occluded objects, we introduce an autoencoder structure in the auxiliary model, as shown in **Figure 2b**. Specifically, the auxiliary model receives images that have been randomly occluded, which are first processed through the first and third feature extraction layers C3K2 of the model. Subsequently, the main model and the auxiliary model perform feature fusion through the Adaptive Gate Information Integration Network (AGIIN, as shown in **Figure 2c**, and the fused features will serve as the input for the next layer of the main model. At this stage, the main model extracts features from the original images, while the auxiliary model extracts features from the occluded images. Next, the branch of the auxiliary model repairs the image through the Convolutional Autoencoder (CAE). Once the repair is complete, the main and auxiliary models perform feature fusion again via the Adaptive Gate Information Integration Network (AGIIN) after the second and fourth C3K2 feature extraction layers, with the fused features serving as input for the subsequent layers of each model. This design enables the main model to learn and absorb the image repair capability of the auxiliary model, thereby significantly improving the model’s performance in detecting occluded objects.

#### 2.1.1 Convolutional Autoencoder

This study presents a Convolutional Autoencoder (CAE), with its architecture described in **Figure 2b**. This model consists of two core components: an encoder and a decoder. It is designed toeffectively compress and accurately reconstruct data by deeply learning the intrinsic features ofthe input data, making it suitable for processing and repairing occluded images. The detailed stepsof this process are described in **Algorithm 1**. The encoder is responsible for extracting features from the input feature map *x*, which has dimensions *H* × *W* × *D*, through a series of convolutional layers and nonlinear activation functions, ultimately generating a low-dimensional encoded representation *z*. This process not only captures the key information of the input data but also lays the foundation for subsequent reconstruction. The decoder is tasked with remapping the encoded representation *z* back to the original data dimensions, gradually restoring a reconstructed feature map $\hat{x}$ similar to the input feature map through a series of transposed convolutional layers and nonlinear activation functions. The advantage of this structure lies in its ability to automatically learn patterns in the data and utilize these patterns during the reconstruction process to fill in the missing information due to occlusions. In this way, the Convolutional Autoencoder not only achieves efficient compression of images but also demonstrates excellent performance in image repair tasks, especially when dealing with occluded and damaged images, significantly improving the accuracy and efficiency of repair.

Algorithm 1 **Convolutional autoencoder for feature inpainting.**

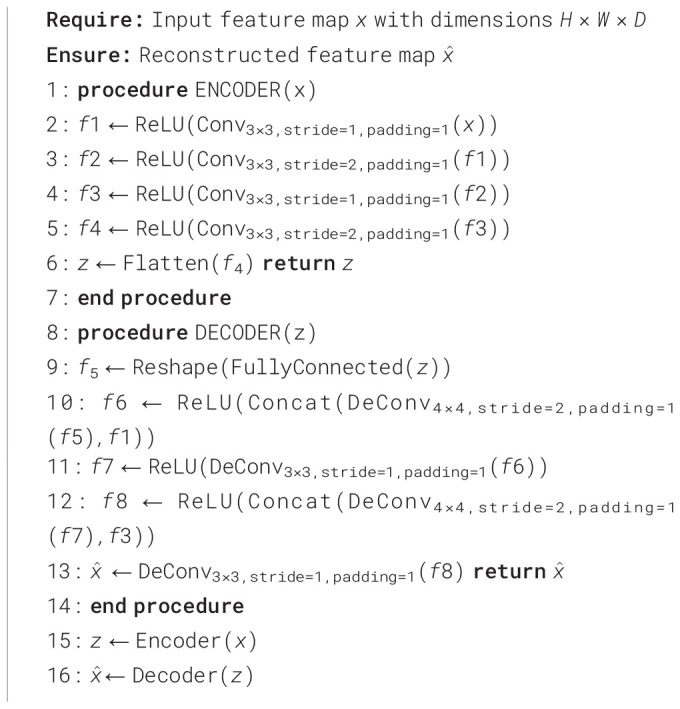

#### 2.1.2 Adaptive Gate Information Integration Network

In this study, we introduce an innovative component—the Adaptive Gate Information Integration Network (AGIIN), which plays a crucial role in the multi-task learning framework, responsible for coordinating the flow of information between different task branches, as shown in **Figure 2c**. AGIIN rigorously controls feature interactions across task branches, filtering out ineffective data and transmitting only critical information to preserve model performance. In implementation, each adaptive gate module calculates the message passing weights between branches through the network connection layer. The computed weights dynamically integrate features across branches, yielding enhanced and task-specific feature representations:

(1) $$\alpha_{k-1}^{xy}=\text{Sigmoid}(M_{k-1}^{x}{\text{f}}_{k-1}^{x}+{\text{a}}_{k-1}^{x}),$$

where $\alpha_{k-1}^{xy}$ represents the information flow coefficient from branch *x* to branch $y,\;f_{k-1}^{x}$ is the output feature of branch x at layer $k-1,\;M_{k-1}^{x}$ and $a_{k-1}^{x}$ are learnable weights and biases, respectively, and Sigmoid is the Sigmoid activation function. Using these weights and the features from the previous layer, we calculate the output feature of branch *y* at the current layer:

(2) $${\text{f}}_{k}^{y}=\alpha_{k-1}^{xy}⊙\text{GELU}(N_{k-1}^{x}{\text{f}}_{k-1}^{x}+{\text{b}}_{k-1}^{x})⊕{\text{f}}_{k-1}^{y},$$

where ⊙ denotes element-wise multiplication, ⊕ denotes element-wise addition, GELU represents the Gaussian Error Linear Unit activation function, $N_{k-1}^{x}$ and ${\text{b}}_{k-1}^{x}$ are learnable weights and biases, respectively, and ${\text{f}}_{k-1}^{y}$ is the output feature of branch *y* at layer k−1. ${\text{f}}_{k}^{y}$ is the output of AGIIN and serves as the input for the next layer of branch *y*. During training, the weights and biases of the network connection layer are automatically updated according to the gradients of the loss function. This adaptive learning capability enables the model to continuously optimize its parameters to adapt to changing data distributions and task requirements.

The theoretical superiority of AGIIN over traditional linear fusion methods (e.g., summation) lies in its capability for dynamic noise suppression via this adaptive gating mechanism. As shown in Equations 1 and 2, the gating weights $\alpha_{k-1}^{xy}$ are constrained within [0,1] via the Sigmoid function, enabling the model to effectively learn to “gate out” background noise or reconstruction artifacts from the auxiliary branch that do not align with the detection task. This learned selectivity is visually substantiated by our Grad-CAM analysis, which demonstrates that the gating mechanism effectively guides the model to focus exclusively on valid pest-related features by assigning higher weights to informative regions while suppressing irrelevant noise.

#### 2.1.3 Adaptive Gate Information Integration Network-assisted Main-auxiliary Model Framework

During the training phase, our method employs a dual-branch structure, including a main model and an auxiliary model, as detailed in **Figure 2a**. This approach aims to enhance the model’s ability to detect occluded objects without increasing the model’s parameter quantity. The main model remains unchanged and directly receives the original images as input. The auxiliary model receives images that have been randomly occluded to simulate special conditions that may occur in real environments, and integrates a Convolutional Autoencoder (CAE) after the first and third feature extraction layers C3K2, as described in 2.1.1.

Firstly, the main model and the auxiliary model receive the same image input, but the input to the auxiliary model is processed with random occlusion. After passing through the first feature extraction layer C3K2, the two models perform the first feature information exchange. This process is realized through the Adaptive Gate Information Integration Network (AGIIN), as detailed in 2.1.2. To ensure the effectiveness of the CAE, during the first feature information exchange, only the feature information from the auxiliary model is passed to the main model to prevent the CAE from prematurely accessing the repaired target features. The first feature information exchange is as follows:

(3) $$\begin{array}{c} {\text{f}}_{k}^{main}=\text{Sigmoid}(M_{k-1}^{aux}{\text{f}}_{k-1}^{aux}+{\text{a}}_{k-1}^{aux})⊙\\ \text{GELU}(N_{k-1}^{aux}{\text{f}}_{k-1}^{aux}+{\text{b}}_{k-1}^{aux})⊕{\text{f}}_{k-1}^{main}, \end{array}$$

where ${\text{f}}_{k}^{main}$ represents the main model feature after feature information exchange, serving as the input for the *k*-th layer of the main model, ${\text{f}}_{k-1}^{aux}$ represents the output feature of the auxiliary model at layer k−1, and $f_{k-1}^{main}$ represents the output feature of the main model at layer k−1. $M_{k-1}^{aux},N_{k-1}^{aux},{\text{a}}_{k-1}^{aux}$, and ${\text{b}}_{k-1}^{aux}$ are trainable weights and biases, respectively, which are updated simultaneously when the main and auxiliary models are updated. Subsequently, the auxiliary model reconstructs the features through the CAE and, after passing through the second feature extraction layer C3K2, the main model and the auxiliary model perform the second feature information exchange. In this exchange, the feature information of the main model is passed to the auxiliary model, and at the same time, the feature information of the auxiliary model is also passed back to the main model. The input feature for the next layer of the main model is calculated in the same way as Equation 3, while the input feature for the next layer of the auxiliary model is calculated as follows:

(4) $$\begin{array}{c} {\text{f}}_{k}^{aux}=\text{Sigmoid}(M_{k-1}^{main}{\text{f}}_{k-1}^{main}+{\text{a}}_{k-1}^{main})⊙\\ \text{GELU}(N_{k-1}^{main}{\text{f}}_{k-1}^{main}+{\text{b}}_{k-1}^{main})⊕{\text{f}}_{k-1}^{aux}, \end{array}$$

where $f_{k}^{aux}$ is the auxiliary model feature after feature information exchange, used as the input for layer k, and $M_{k-1}^{main},N_{k-1}^{main},a_{k-1}^{main}$, and $b_{k-1}^{main}$ are trainable weights and biases, respectively, which are updated simultaneously when the main and auxiliary models are updated. The meanings of the other symbols are consistent with the previous text.

These two rounds of feature information exchange enable the main model to integrate original image features, occluded image features, and reconstructed occluded image features, thereby acquiring a certain ability to detect occluded objects without relying on the CAE. At the same time, in the second feature information exchange, the auxiliary model receives the original image feature information passed from the main model, which helps guide the direction of image reconstruction by the auxiliary model. Since the feature information exchange is conducted through AGIIN, it does not affect the performance of the main model on the original task due to the integration of too many features from the auxiliary model.

Furthermore, to enhance the robustness of the main model in detecting occluded objects, we repeated the above feature information exchange process in the third and fourth feature extraction layers C3K2 of the model. This strategy further strengthens the model’s adaptability to occlusions, ensuring efficient performance in complex environments.The information exchange is performed exactly twice, after the 1st/2nd and 3rd/4th C3K2 layers in the backbone, respectively. This design is constrained by the YOLO11n architecture, which contains only four major feature extraction stages (C3K2 blocks) suitable for crossbranch fusion before entering the neck.The rationale for designating these C3K2 layers as exchange nodes is rooted in the hierarchical feature extraction process of the backbone. As the primary units for representation learning, C3K2 modules capture essential multi-scale spatial information. By implementing exchange at these fundamental stages, the auxiliary model provides fine-grained guidance to the primary model’s feature maps before they undergo high-level semantic fusion in the Neck section. This strategy ensures that “refined” features effectively enhance the main model’s perception of small and occluded objects, as evidenced by the substantial performance gains reported in **Table 1**. This dual-iteration configuration is considered optimal for the YOLO11n architecture as it fully utilizes all available backbone stages without introducing excessive computational overhead or requiring structural modifications to the original network.Additional exchanges are not feasible without structural modifications.

**Table 1:** Comparison of experimental results using our dataset.

Without Occlusion	Prec.	Rec.	mAP50	mAP50-95	Params.	GFLOPs(train)	GFLOPs(test)
YOLO11n	83.3	82.1	86.8	56.5	2,624,064	6.6	6.6
RT-DETR-L	81.2	79.7	83.2	52.6	33,003,356	108.4	103.9
EfficientDet-d2	86.4	85.6	88.1	56.7	8,010,594	11.1	10.7
Mask-RCNN	80.2	73.7	79.4	49.6	44,821,294	116.1	112.4
YOLOv12n	84.5	82.8	87.9	57.5	2,557,508	6.3	6.3
AGIIN-MAF	83.1	76.9	84.4	53.8	5,864,088	108.6	6.6
With Occlusion	Prec.	Rec.	mAP50	mAP50-95	Params.	GFLOPs(train)	GFLOPs(test)
YOLO11n	74.2	55.9	64.1	32.5	2,624,064	6.6	6.6
RT-DETR-L	77.1	64.6	71.3	39.1	33,003,356	108.4	103.9
EfficientDet-d2	65.2	73.1	70.7	42.1	8,010,594	11.1	10.7
Mask-RCNN	63.7	61.2	60.4	35.7	44,821,294	116.1	112.4
YOLOv12n	69.2	63.1	67.5	35.5	2,557,508	6.3	6.3
AGIIN-MAF	81.8	72.4	80.2	50.1	5,864,088	108.6	6.6

Methods with references: YOLO11n Jocher et al. (2023), RT-DETR-L Zhao et al. (2024b), EfficientDet-d2 Tan et al. (2020), Mask-RCNN He et al. (2017), YOLOv12n Tian et al. (2025).

To guide the model to develop in the direction we expect, we update the model by taking into account the losses of both the main model and the auxiliary model after each batch. The total loss is calculated as follows:

(5) $$L_{t}=L_{main}+\gamma \times L_{aux}$$

In this formula, $L_{t}$represents the total loss of the model, $L_{main}$ represents the loss of the main model, $L_{aux}$represents the loss of the auxiliary model, and *γ* is a loss weight coefficient used to adjust the proportion of the auxiliary model loss in the total loss. During the model update process, we update not only the parameters of the main model and the auxiliary model but also the parameters of the CAE and AGIIN. The specific update process is as follows:

(6) $$\theta_{main}\leftarrow \theta_{main}-\epsilon \nabla_{\theta_{main}}L_{t}$$

(7) $$\theta_{aux}\leftarrow \theta_{aux}-\epsilon \nabla_{\theta_{aux}}L_{t}$$

(8) $$\theta_{CAE}\leftarrow \theta_{CAE}-\epsilon \nabla_{\theta_{CAE}}L_{t}$$

(9) $$\theta_{AGIIN}\leftarrow \theta_{AGIIN}-\epsilon \nabla_{\theta_{AGIIN}}L_{t}$$

Here, $\theta_{main},\theta_{aux},\theta_{CAE}$, and $\theta_{AGIIN}$ represent the parameters of the main model, auxiliary model, CAE, and AGIIN, respectively. $\nabla_{\theta_{main}},\nabla_{\theta_{aux}},\nabla_{\theta_{CAE}}$, and $\nabla_{\theta_{AGIIN}}$ represent their respective gradients, and *ϵ* is the learning rate during training.

This update strategy balances the losses of the main and auxiliary models, thereby improving the overall performance and generalization ability of the model.

### 2.2 Testing phase

#### 2.2.1 Parameter sensitivity assessment

**Figure 3:**
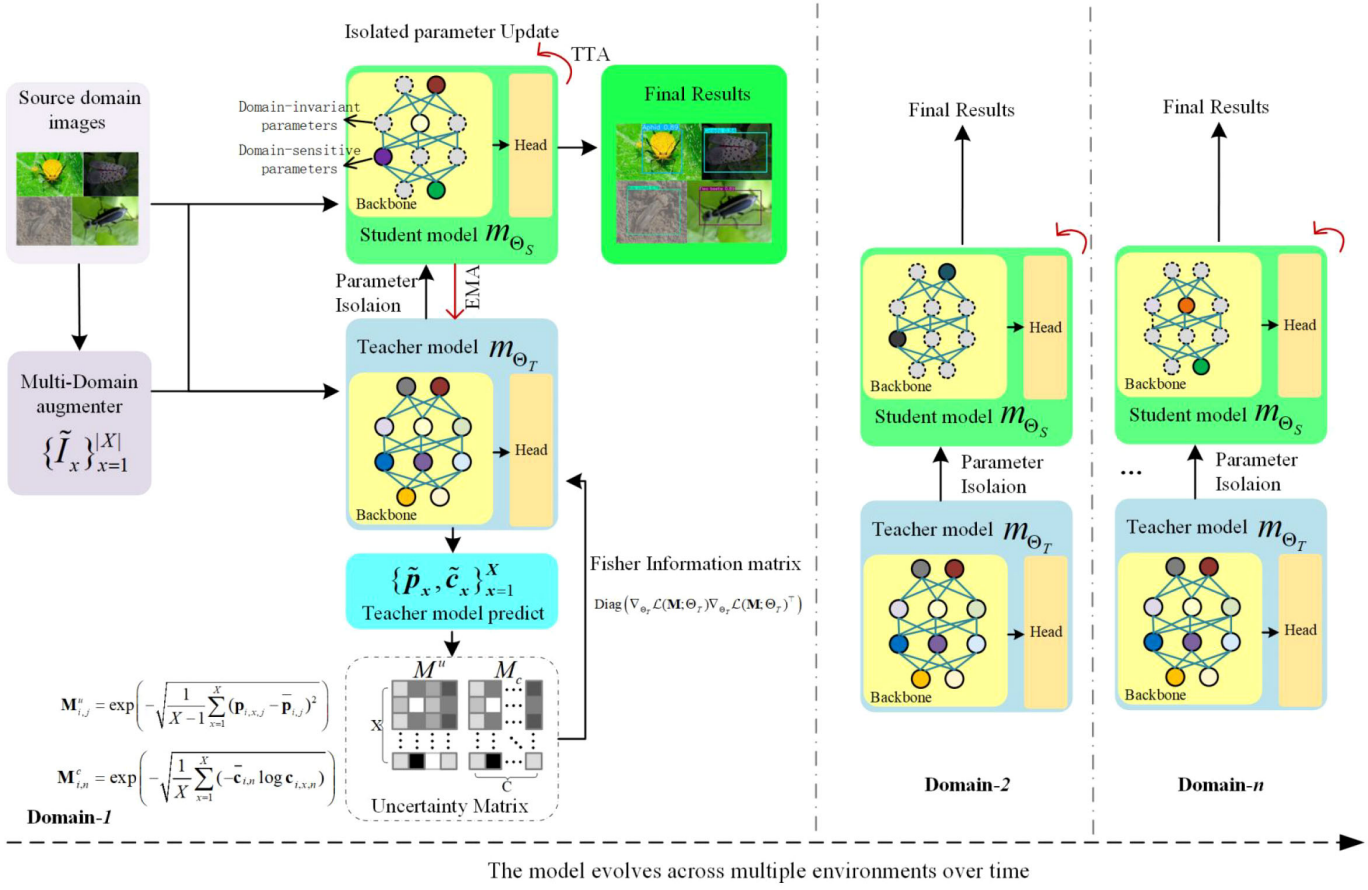
Detailed flowchart of the SAODL framework. This framework employs multi-domain augmentation to process source domain images, generating a set of enhanced images. These images are fed into the teacher model, which produces predictions. These predictions are then compared with those from the original image to compute uncertainty matrices for bounding boxes and categories. The Fisher Information Matrix is utilized to categorize model parameters into domain-invariant and domain-sensitive parameters. In SAODL, domain-sensitive parameters of the student model are updated based on uncertainty matrices. Simultaneously, the teacher model undergoes isolated exponential moving average (EMA) updates to ensure stability of domain-invariant parameters. This process is iterated across multiple domains, ensuring that the model adapts to new domains while preserving its generalization ability, ultimately yielding robust final results.

After training, the auxiliary model, convolutional autoencoder (CAE), and all random occlusion augmentations are discarded to ensure lightweight and real-time inference. Only the trained main model is deployed.To enable continuous adaptation to unseen and dynamically changing environments, we propose the Selective Adaptive Optimization for Domain-aware Learning (SAODL) strategy, whose detailed logical flow is depicted in **Figure 3**, which operates in a teacher-student framework using two copies of the main model: a teacher model updated via masked exponential moving average (EMA) and a student model with selective parameter updates. Importantly, both the teacher and student receive the same original test image without any artificial occlusion, eliminating the extra branches and computational overhead introduced solely for training.

In this section, we employ a novel image processing technique aimed at investigating the sensitivity of model parameters during domain adaptation. Domain adaptability analysis, also known as uncertainty quantification, is an important means in the field of machine learning to measure the robustness of a model. This analysis plays an extremely critical role in ensuring that the model can still maintain good performance when the data distribution changes, especially in situations where data acquisition is difficult, costly, or limited in quantity.

In our research, the model does not have access to source data during the testing phase, so it needs to adapt to the target domain without the aid of additional training data. Based on this, inspired by current research on uncertainty quantification, we propose a new method of domain adaptability analysis, which aims to measure the sensitivity of model parameters to the new target domain.

The key point of this method is to divide the model parameters into two major parts: domain-invariant parameters and domain-sensitive parameters. Domain-invariant parameters reflect the knowledge that the model can transfer between different domains, while domain-sensitive parameters capture the unique information of a specific domain. Retaining domain-invariant parameters ensures generalizability, while updating domain-sensitive parameters optimizes the model’s alignment with the target domain.

Traditional uncertainty quantification methods are based on pre-trained models that perform well in the source domain to evaluate the confidence of predictions. However, when the model adapts to the constantly changing target domain, these methods may become unstable and inaccurate in evaluating confidence. To address this, we adopt the homeostasis mechanism from biological systems—a self-regulating process that ensures stability and quantifies sensitivity to external perturbations.

Specifically, for a given original image **I** and its corresponding series of augmented images ${\left\{{\tilde{I}}_{x}\right\}}_{x=1}^{\left|X\right|}$, where X represents the total number of augmented images, we first input them into the teacher model *m*_Θ_*_T_*to obtain object bounding box predictions ${\left\{{\tilde{p}}_{x}\right\}}_{x=1}^{X}$ and object categories ${\left\{{\tilde{c}}_{x}\right\}}_{x=1}^{X}$.These augmented views are generated through a stochastic combination of geometric and photometric transformations to simulate the diverse visual perturbations encountered in real-world agricultural settings. By aggregating prediction differences across X=8 distinct views, the resulting uncertainty matrix captures a robust estimate of parameter sensitivity that is less dependent on any specific transformation, ensuring stable adaptation even under complex distribution shifts as demonstrated in our experimental results. We refer to the enhanced bounding boxes and object category predictions as intermediate labels, while the predictions of the original image are called proxy labels.

On the other hand, the original **I** is also input into the model *m*_Θ_*_T_* to obtain proxy labels, that is, object bounding box predictions $\hat{p}$ and object categories $\hat{c}$. Subsequently, we calculate the differences between the intermediate bounding boxes ${\left\{{\tilde{p}}_{x}\right\}}_{x=1}^{X}$ and the proxy bounding box $\hat{p}$, forming *X* sets of probability matrices, and take their average as the uncertainty matrix $M^{u}$ of the bounding box. In this study, we mainly focus on the uncertainty matrix related to the four corner coordinates of the bounding box. Each element $M_{i,j}^{u}\in M^{u}$ represents the uncertainty score of the *j*-th corner ( $j\in \{x_{1},y_{1},x_{2},y_{2}\}$) of the *i*-th predicted bounding box, calculated as.

(10) $$M_{i,j}^{u}=\text{exp }(-\sqrt{\frac{1}{X-1}\sum_{x=1}^{X}{\left({\tilde{p}}_{i,x,j}-{\bar{p}}_{i,j}\right)}^{2}})$$

where ${\tilde{p}}_{i,x,j}$denotes the *j*-th corner coordinate of the *i*-th bounding box predicted on the *x*-th augmented view, and ${\bar{p}}_{i,j}$ is the corresponding corner coordinate predicted on the original (unaugmented) image.

To associate bounding boxes across augmented views, we adopt the same object-to-object matching strategy as in common test-time augmentation: for each predicted box in the original image, we find the box with the highest IoU in each augmented view. If no box in an augmented view has IoU ¿ 0.1 with any proxy box, we treat its four corner coordinates as the proxy box coordinates (i.e., zero deviation), which naturally results in high uncertainty and is beneficial for subsequent parameter selection.

Similarly, we calculate the uncertainty matrix **M***^c^* for object categories, where each element $M_{i,n}^{c}\subset M^{c}$ is calculated as follows:

(11) $$M_{i,n}^{c}=\text{exp }(-\sqrt{\frac{1}{X}\sum_{x=1}^{X}(-{\bar{c}}_{i,n}\text{log }c_{i,x,n})}),$$

here, $M_{i,n}^{c}$represents the uncertainty score of the *n*-th category of the *i*-th object, ${\tilde{c}}_{i,x,n}$ is the probability of the *n*-th category predicted for the *i*-th object in the *x*-th augmented image, and ${\bar{c}}_{i,n}$ is the corresponding value in the original image.

Note that the dimension of the uncertainty matrix $M_{u}$ is X×4, that is, the number of augmented images multiplied by the 4 corners of the bounding box; while the dimension of $M_{c}$ is X×C, where C is the total number of object categories. To fully consider the uncertainty, we integrate the uncertainty matrices $M_{u}$ and $M_{c}$to form a unified uncertainty matrix M, defined as follows:

(12) $$\text{M}=[{\text{M}}^{u};{\text{M}}^{c}],$$

where [·;·] denotes the horizontally concatenation of two matrices. Finally, the uncertainty matrix **M** reflects the model’s uncertainty scores for the enhanced bounding boxes and object categories predictions in the new domain, which is then used to assess the parameter sensitivity of the model to the new target domain.

#### 2.2.2 Separation of domain-sensitive and domain-invariant parameters

In this study, we propose an innovative data-driven selective adaptation optimization method aimed at dividing model parameters into two groups: domain-sensitive parameters and domain-invariant parameters, and designing different optimization strategies for these two groups of parameters. The core of this method lies in the use of the Fisher Information Matrix (FIM), a powerful tool extensively employed in uncertainty quantification and parameter sensitivity analysis, to identify the parameters most critical for adaptation to new domains and those that remain relatively stable.

To achieve this goal, we first define the set of parameters for the teacher model ${\text{Θ}}_{T}\in {\mathbb{R}}^{A}$, where *A* represents the total number of model parameters. We assume that the student model has the same architecture and number of parameters, with its parameter set denoted as ${\text{Θ}}_{S}\in {\mathbb{R}}^{A}$. The FIM F(Θ) is defined as the expectation of the outer product of the gradient of the log-likelihood function with respect to the model parameters, specifically as follows:

(13) $$\text{F}(\text{Θ})=E_{I∼D_{T}}[\nabla_{{\text{Θ}}_{T}}\text{log }p(\text{M}|I;{\text{Θ}}_{T})\nabla_{{\text{Θ}}_{T}}\text{log }p{\left(\text{M}|I;{\text{Θ}}_{T}\right)}^{⊤}],$$

where M represents the output of the model, and D_T_ is the distribution of the training data.

To simplify the computation and enhance the interpretability of the results, we assume that the model parameters are independent and only use the diagonal elements of the FIM to approximate the importance of the parameters, defining the approximate matrix **K**(Θ) as follows:

(14) $$\text{K}(\text{Θ})=\text{diag}(\nabla_{{\text{Θ}}_{T}}L(M;{\text{Θ}}_{T})\nabla_{{\text{Θ}}_{T}}L{\left(M;{\text{Θ}}_{T}\right)}^{⊤}),$$

where L denotes the loss function of the model, and the diag(·) operation extracts the diagonal elements of the matrix. Each element of K(Θ) corresponds to an importance metric of a model parameter, with larger values indicating greater sensitivity of the parameter to domain changes.

To distinguish between parameters sensitive and invariant to new domains within the model parameters, we introduce a binary mask $b\in {\left\{0,1\right\}}^{A}$, with elements following a Bernoulli distribution:

(15) $${\text{b}}_{\text{i}}∼\text{Bernoulli}(\alpha ),\;i=1,\ldots ,A,$$

where *α* is the probability of taking the value 1. Based on **K**(Θ), we set the elements of the mask **b** as follows:

(16) $${\text{b}}_{\text{i}}=\{\begin{array}{ll} 1, & \text{if}\;K(\theta_{i})>\tau \\ 0, & \text{otherwise} \end{array},$$

where *τ* is a preset threshold used to differentiate the importance of parameters. In the experiments, we set *τ* to 0.3.In our implementation, *τ* is set to 0.3. This selection is based on the empirical distribution of normalized Fisher information values observed in preliminary tests, serving as an optimal cut-off point to maintain the stability-plasticity balance. Specifically, this threshold ensures that the model preserves enough source knowledge (stability) while allowing sufficient flexibility for domain-sensitive adaptation (plasticity). The robustness of this setting is substantiated by the consistent performance gains across three different agricultural datasets (**Tables 1**–**3**), which indicates that the model is not overly sensitive to this specific parameter choice across various environments. In this way, we can identify parameters sensitive to changes in the new domain (**b***_i_*= 1) and domain-invariant parameters (**b***_i_*= 0), and adjust the optimization strategy accordingly.

**Table 2:** Comparison of experimental results using IP102 dataset (/%).

Without Occlusion	Prec.	Rec.	mAP50	mAP50-95	Params.	GFLOPs(train)	GFLOPs(test)
YOLO11n	50.4	57.9	55.2	36.1	2,624,064	6.6	6.6
RT-DETR-L	**57.3**	54.5	51.7	32.5	33,003,356	108.4	103.9
EfficientDet-d2	55.7	58.3	54.6	35.4	8,010,594	11.1	10.7
Mask-RCNN	51.4	52.6	49.2	30.3	44,821,294	116.1	112.4
YOLO12n	53.2	57.0	55.3	36.0	**2,557,508**	**6.3**	**6.3**
AGIIN-MAF	51.7	**58.4**	**56.6**	**36.8**	5,864,088	108.6	6.6
With Occlusion	Prec.	Rec.	mAP50	mAP50-95	Params.	GFLOPs(train)	GFLOPs(test)
YOLO11n	40.5	40.1	39.4	23.2	2,624,064	6.6	6.6
RT-DETR-L	**50.1**	32.2	33.1	16.9	33,003,356	108.4	103.9
EfficientDet-d2	42.1	50.2	44.1	26.3	8,010,594	11.1	10.7
Mask-RCNN	39.2	42.1	31.1	15.7	44,821,294	116.1	112.4
YOLO12n	45.1	39.7	38.2	20.7	**2,557,508**	**6.3**	**6.3**
AGIIN-MAF	47.1	**50.9**	**51.2**	**32.1**	5,864,088	108.6	6.6

**Table 3:** Comparison of experimental results using pest detection dataset (/%).

Without occlusion	Precision	Recall	mAP50	mAP50-95
YOLO11n	78.6	63.6	70.7	36.1
RT-DETR-L	79.9	62.2	65.4	33.7
EfficientDet-d2	82.7	67.2	72.9	37.3
Mask-RCNN	76.6	58.3	63.6	32.4
YOLO12n	83.8	57.2	70.7	36.4
AGIIN-MAF	78.7	62.9	70.4	35.8
With Occlusion	Precision	Recall	mAP50	mAP50-95
YOLO11n	58.9	54.0	56.5	26.8
RT-DETR-L	66.5	49.6	53.3	26.1
EfficientDet-d2	62.7	57.4	58.6	28.2
Mask-RCNN	58.2	48.1	50.4	23.6
YOLO12n	62.5	49.6	57.3	27.4
AGIIN-MAF	74.8	57.2	65.5	32.4

This method not only enhances the model’s adaptability to new domains but also reduces unnecessary computational overhead and improves training efficiency by precisely controlling parameter updates. Experimental verification shows that this method performs excellently in multiple domain adaptation tasks, significantly improving the performance and stability of the model.

#### 2.2.3 Selective adaptive optimization for domain-aware learning

To maintain the model’s generalization capacity, we propose categorizing the model parameters into two groups: domain-sensitive parameters and domain-invariant parameters. Furthermore, we introduce a Selective Adaptive Optimization for Domain-aware Learning (SAODL) strategy, aimed at assigning different optimization methods to these two groups of parameters, namely isolated parameter optimization for the student model and Exponential Moving Average (EMA) update for the teacher model.SAODL follows a teacher-student framework with isolated updates: only domain-sensitive parameters (identified by large Fisher values) are updated in the student using pseudo-labels from the teacher, while domain-invariant parameters are preserved via a masked EMA. This prevents catastrophic forgetting of general object detection knowledge while allowing fast adaptation to domain-specific shifts (lighting, weather, blur, etc.).

Isolated Parameter Optimization for the Student Model.

Given the binary mask b, which identifies the domain-sensitive weights and stable weights in the teacher model Θ*_T_*, we apply this mask to the student network Θ*_S_* to isolate the corresponding parameters. For a sample $I^{\left(t\right)}$ from the new domain, it is fed into the unoptimized student and teacher networks from the previous step *t* − 1, namely ${\text{Θ}}_{S}^{\left(t-1\right)}$ and ${\text{Θ}}_{T}^{\left(t-1\right)}$, to produce predictions $\left[{\tilde{p}}_{S}^{\left(t\right)},{\tilde{c}}_{S}^{\left(t\right)}]={\text{Θ}}_{S}^{\left(t-1\right)}(I^{\left(t\right)}\right)$ and $\left[{\tilde{p}}_{T}^{\left(t\right)},{\tilde{c}}_{T}^{\left(t\right)}]={\text{Θ}}_{T}^{\left(t-1\right)}(I^{\left(t\right)}\right)$. Here, $\left[{\tilde{p}}_{T}^{\left(t\right)},{\tilde{c}}_{T}^{\left(t\right)}\right]$ serves as the pseudo-labels for the bounding box ${\tilde{p}}_{T}^{\left(t\right)}$and object category ${\tilde{c}}_{T}^{\left(t\right)}$. Subsequently, we update only the domain-sensitive parameters ${\text{Θ}}_{S}^{\left(t-1\right)}⊗\text{b}$ to obtain the adapted parameters ${\text{Θ}}_{S}^{\left(t\right)}$ for the student network. This process is realized through a specific gradient descent step, defined as follows:

(17) $${\text{Θ}}_{S}^{\left(t\right)}\leftarrow {\text{Θ}}_{S}^{\left(t-1\right)}-\eta \nabla_{{\text{Θ}}_{S}^{\left(t-1\right)}}L_{det}([{\tilde{p}}_{S}^{\left(t\right)},{\tilde{c}}_{S}^{\left(t\right)}],[{\tilde{p}}_{T}^{\left(t\right)},{\tilde{c}}_{T}^{\left(t\right)}])⊗\text{b},$$

where *η* = 0.001 is the learning rate of the student model, ⊗ denotes element-wise multiplication, and $\nabla_{{\text{Θ}}_{S}^{\left(t-1\right)}}$ is the gradient with respect to the student model parameters.This value was empirically determined through preliminary tests to ensure a stable adaptation process, preventing the model from over-fitting to potential noise in the pseudo-labels generated under extreme environmental interference. Additionally, the detection loss function $L_{det}$is defined as the weighted sum of the classification loss $L_{cls}$, bounding box regression loss $L_{bb}$, and confidence loss $L_{conf}$:

(18) $$L_{det}=\lambda_{cls}L_{cls}+\lambda_{bb}L_{bb}+\lambda_{conf}L_{conf},$$

where $\lambda_{cls},\lambda_{bb}$, and $\lambda_{conf}$ are hyperparameters.

Through this isolated parameter update strategy, the student model can adapt to the new target domain, where the domain-sensitive parameters ${\text{Θ}}_{S}^{\left(t-1\right)}⊗\text{b}$ are updated to capture the specific distribution changes within the target domain. Meanwhile, the domain-invariant parameters ${\text{Θ}}_{S}^{\left(t-1\right)}⊗(1-b)$ are retained to maintain the model’s generalization ability, prevent catastrophic forgetting, and bring long-term benefits to the model’s adaptation. Once the adapted student model ${\text{Θ}}_{S}^{\left(t\right)}$ is obtained, forward propagation is performed to predict the final detection bounding boxes and categories $\left[p_{S}^{\left(t\right)},c_{S}^{\left(t\right)}]={\text{Θ}}_{S}^{\left(t\right)}(I^{\left(t\right)}\right)$.

Isolated Exponential Moving Average for the Teacher Model.

In standard knowledge distillation or test-time adaptation models, there is often the drawback of updating the entire set of model parameters, where some key parameters may be forgotten during the optimization process of Exponential Moving Average (EMA):

(19) $${\text{Θ}}_{T}^{\left(t\right)}\leftarrow \mu {\text{Θ}}_{T}^{\left(t-1\right)}+(1-\mu ){\text{Θ}}_{S}^{\left(t\right)},$$

where ${\text{Θ}}_{T}^{\left(t\right)}$ is the updated teacher network, μ=0.85 is the momentum factor. ${\text{Θ}}_{S}^{\left(t\right)}$ is the adapted student network, and ${\text{Θ}}_{T}^{\left(t-1\right)}$ is the previous teacher network.The selection of μ=0.85 balances the integration of new domain knowledge from the student model with the preservation of foundational knowledge in the teacher model, ensuring a robust stability-plasticity trade-off as evidenced by the consistent results across various datasets. We note that the standard EMA fails to selectively optimize model parameters, which may lead to a decline in model performance.

Therefore, in the case of a constantly changing target domain, it is necessary to isolate the parameters of the teacher model to retain domain-generalizable knowledge. To achieve this, we propose an isolated EMA optimization strategy to update the teacher model Θ*_T_*, defined as follows:

(20) $${\text{Θ}}_{T}^{\left(t\right)}\leftarrow {\text{Θ}}_{T}^{\left(t-1\right)}⊗b+(1-b)⊗(\mu {\text{Θ}}_{T}^{\left(t-1\right)}+(1-\mu ){\text{Θ}}_{S}^{\left(t\right)}).$$

This equation advances the classical EMA strategy to selectively update the domain-sensitive parameters ${\text{Θ}}_{T}^{\left(t-1\right)}⊗\text{b}$ and maintain the other invariant parameters $\left(1-\text{b})⊗(\mu {\text{Θ}}_{T}^{\left(t-1\right)}+(1-\mu ){\text{Θ}}_{S}^{\left(t\right)}\right)$. We note that the proposed isolated EMA is a simple and easily implementable optimization method without additional computational costs.

After the student and teacher models are updated, for the next image **I**^(^*^t^* ^+ 1)^, the model adaptation begins with ${\text{Θ}}_{S}^{\left(t+1\right)}\leftarrow {\text{Θ}}_{S}^{\left(t\right)}$ and ${\text{Θ}}_{T}^{\left(t+1\right)}\leftarrow {\text{Θ}}_{T}^{\left(t\right)}$ as initial parameters, and the above process is repeated. Our isolated student and teacher model optimization strategies can adapt to the constantly changing target domain and prevent catastrophic forgetting of source domain knowledge, thereby enhancing the model’s generalization ability in dynamically evolving detection scenarios.

## 3 Experiments

### 3.1 Datasets and experimental settings

In our experiments, to ensure the reliability of the results, we utilized a dataset that combined a portion of the filtered IP102 dataset (Wu et al., 2019) with our self-collected dataset. The resulting dataset consists of 10,916 training set images, 2,001 validation set images, and 673 testing set images, which were used for training, validation, and testing during the training phase. This dataset covers 40 pest categories, with each image sized at 640 pixels. To validate the effectiveness of the model, we prepared multiple datasets, including the Pest Detection Dataset, Pest Vision Major Dataset, IP102, and Forestry Pest Dataset (Liu et al., 2022). These datasets are available in both YOLO and COCO formats to meet the requirements of different model training and validation. Within this experimental setup, the testing set images were randomly occluded to evaluate the model’s capability to detect occluded objects. Moreover, to assess the impact of test-time adaptation, we prepared nine types of corrupted images—fog, rain, snow, bright, dark, Gaussian noise, salt and pepper noise, motion blur, and JPEG compression—with 673 images per type, amounting to a total of 6,057 images. These served as the test set for evaluating test-time adaptation performance.To further evaluate cross-dataset generalization, it is essential to recognize the significant distributional shifts among the five utilized datasets. For instance, the IP102 dataset primarily consists of high-resolution images with relatively clean backgrounds, whereas the Forestry Pest dataset contains complex forest canopies with dramatic shadow and lighting variations. Our self-collected Pest Detection Dataset specifically focuses on small-scale pests in dynamic field environments. These datasets represent a wide spectrum of domain gaps: (1) Background complexity (ranging from simple laboratory backgrounds to dense agricultural foliage). (2) Illumination variance (from controlled indoor lighting to unpredictable outdoor sunlight). And (3) Scale diversity (from large, centered individuals to tiny, clustered pests).

All experiments were conducted on a workstation equipped with an NVIDIA GeForce RTX 4090 (24GB) GPU and an Intel Core i9-13900K processor. During the training phase, the initial learning rate was set to *lr*0 = 0.015, the final learning rate scaling factor to *lrf* = 0.01, and the learning rate *ϵ* was dynamically adjusted with the epoch. The training was set to *epochs* = 50, *batch size* = 16. In the testing phase, the teacher model received *N* = 8 augmented images, the student model learning rate was *η* = 0.001, and the teacher model EMA momentum factor was *µ* = 0.85.To ensure a fair and rigorous comparison, it is imperative to note that the proposed AGIIN-MAF model and all baseline comparison models (i.e., YOLO11n, RT-DETR-L, EfficientDet-d2, Mask-RCNN, and YOLOv12n) were trained entirely under identical experimental protocols. Specifically, all models utilized the exact same hyperparameters mentioned above, identical multi-domain data augmentation strategies including random occlusion, and were independently executed on the same hardware environment to minimize random variations.

In the experiments, we used the commonly used evaluation metrics of Precision, Recall, mAP50, and mAP50-95.AGIIN-MAF is based on YOLO11n. All reported results are the mean values averaged over 5 independent runs with different random seeds.

### 3.2 Training phase

To comprehensively evaluate the performance of various models across different datasets, we analyzed the experimental results presented in **Tables 1**–**5**, focusing on their ability to handle occlusion and maintain computational efficiency. When examining the custom dataset (**Table 1**) and the IP102 dataset (**Table 2**), a clear pattern emerges regarding occlusion robustness. On the custom dataset, the AGIIN-MAF model demonstrates superior performance, with its mAP50 dropping only from 84.4% to 80.2% under occlusion, compared to YOLO11n’s significant decline from 86.8% to 64.1% and RT-DETR-L’s reduction from 83.2% to 71.3%. Similarly, on the IP102 dataset, AGIIN-MAF’s mAP50 decreases modestly from 56.6% to 51.2% (a 5.4% drop), far outperforming YOLO11n’s 15.8% drop (55.2% to 39.4%) and RT-DETR-L’s 18.6% drop (51.7% to 33.1%). These results underscore AGIIN-MAF’s exceptional resilience in complex occlusion scenarios, attributed to its integration of CAE and AGIIN components, which enhance feature extraction and integration under challenging conditions.

**Table 4:** Comparison of experimental results using pest vision major dataset (/%).

Without occlusion	Precision	Recall	mAP50	mAP50-95
YOLO11n	87.9	86.4	92.0	73.9
RT-DETR-L	88.7	87.5	90.6	74.1
EfficientDet-d2	83.6	91.4	94.2	76.1
Mask-RCNN	86.7	82.3	86.6	68.7
YOLO12n	91.9	88.0	93.1	75.6
AGIIN-MAF	88.5	86.7	91.2	73.5
With Occlusion	Precision	Recall	mAP50	mAP50-95
YOLO11n	78.3	69.7	75.5	52.8
RT-DETR-L	78.7	68.3	74.2	53.0
EfficientDet-d2	70.2	78.1	75.3	56.4
Mask-RCNN	65.1	65.2	70.3	48.4
YOLO12n	73.2	71.7	74.5	50.1
AGIIN-MAF	84.1	78.7	84.6	66.5

**Table 5:** Comparison of experimental results using forestry pest dataset (/%).

Without occlusion	Precision	Recall	mAP50	mAP50-95
YOLO11n	97.0	95.4	98.2	83.4
RT-DETR-L	96.9	98.1	99.1	89.4
EfficientDet-d2	98.4	98.9	98.8	89.6
Mask-RCNN	94.1	92.4	94.7	84.3
YOLO12n	97.8	98.0	99.1	89.3
AGIIN-MAF	96.7	96.2	98.2	86.2
With Occlusion	Precision	Recall	mAP50	mAP50-95
YOLO11n	79.7	64.3	70.5	49.0
RT-DETR-L	85.1	69.0	73.2	53.5
EfficientDet-d2	74.7	84.3	79.4	66.2
Mask-RCNN	71.1	72.3	69.7	55.4
YOLO12n	86.4	70.3	77.0	57.3
AGIIN-MAF	91.7	87.3	91.2	77.8

**Figure 4:**
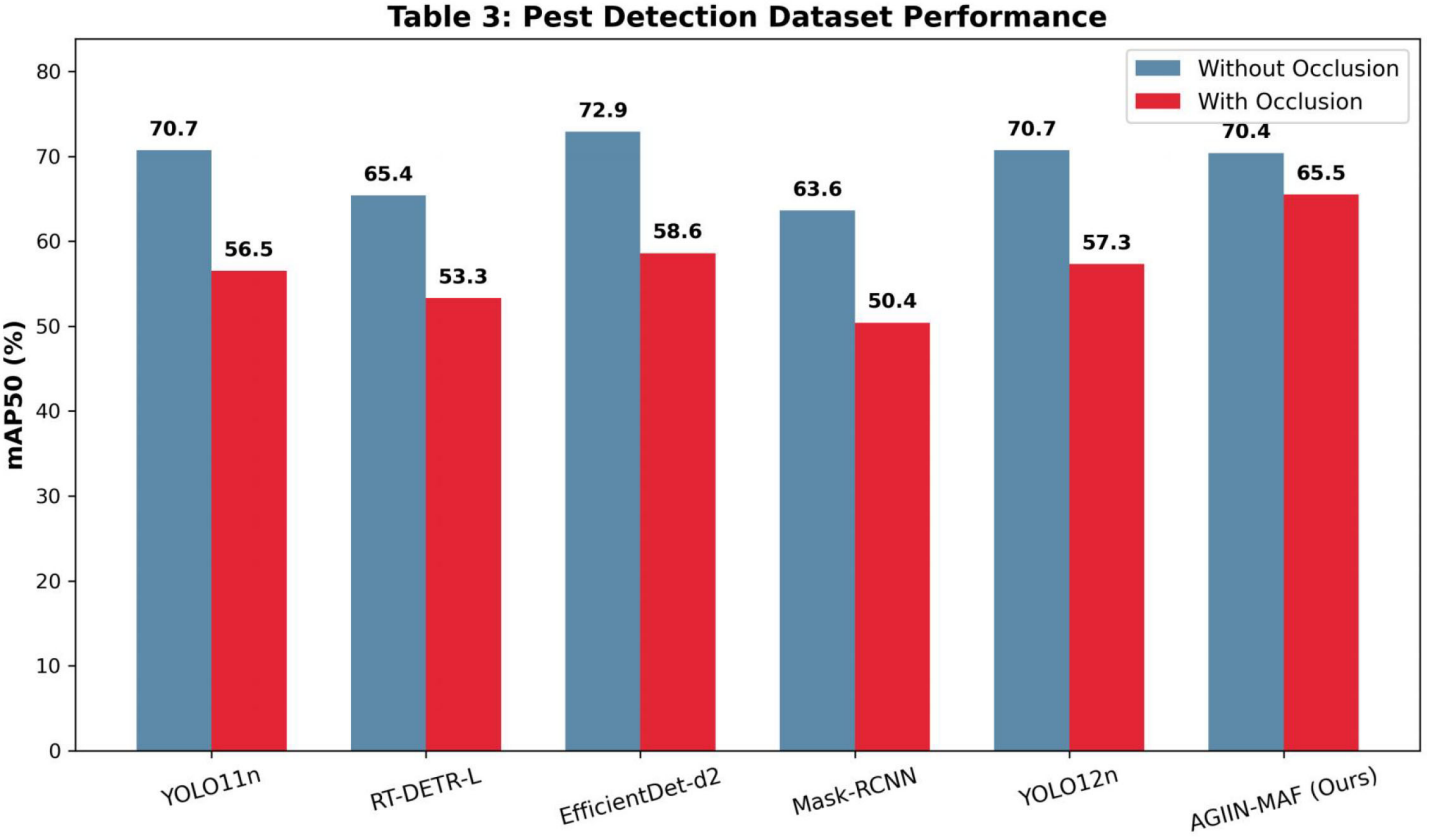
Visual comparison of mAP50 performance on the pest detection dataset.

**Figure 5:**
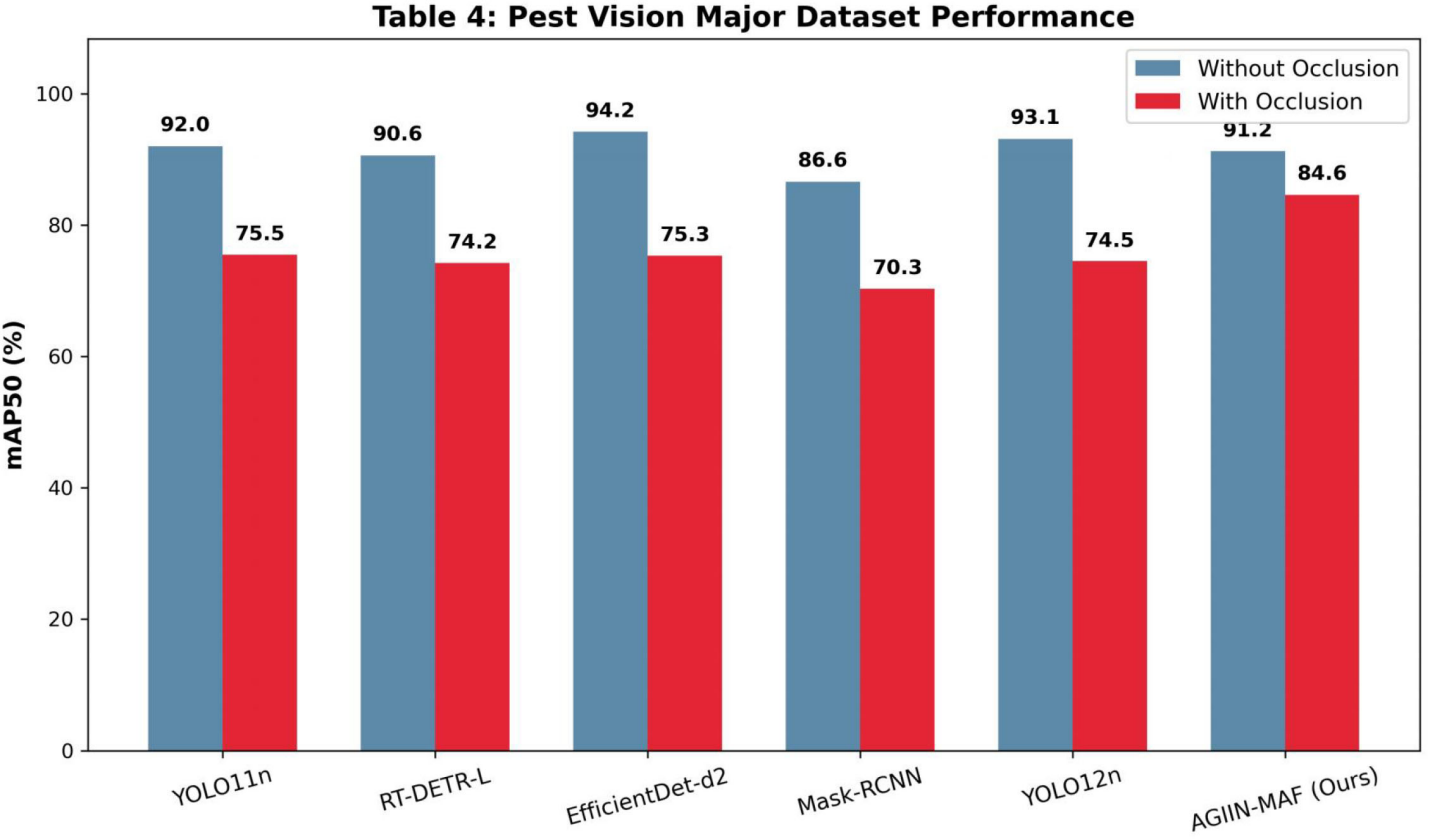
Visual comparison of mAP50 performance on the pest vision major dataset.

**Figure 6:**
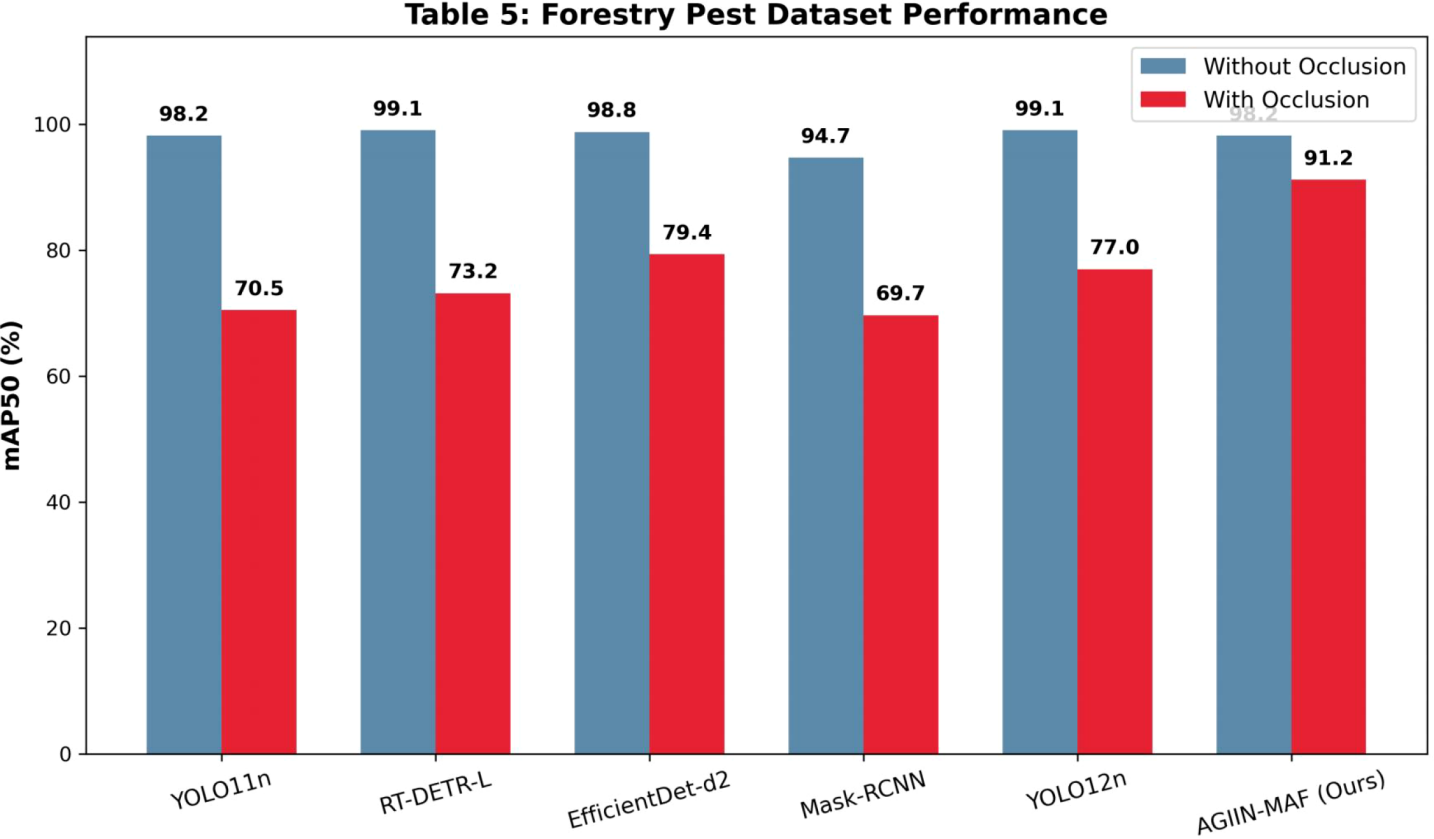
Visual comparison of mAP50 performance on the forestry pest dataset.

Further analysis across the Pest Detection (**Table 3**), Pest Vision Major (**Table 4**), and Forestry Pest (**Table 5**) datasets reveals AGIIN-MAF’s consistent superiority in occlusion scenarios, alongside its generalization capability. On the Pest Detection dataset, AGIIN-MAF achieves an mAP50 of 65.5% with occlusion, surpassing YOLO11n’s 56.5% and RT-DETR-L’s 53.3%, despite starting from comparable non-occluded baselines (70.4%, 70.7%, and 65.4%, respectively). In the Pest Vision Major dataset, AGIIN-MAF’s mAP50 under occlusion reaches 84.6%, significantly higher than YOLO11n’s 75.5% and RT-DETR-L’s 74.2%, maintaining a smaller performance gap from its non-occluded 91.2%. The Forestry Pest dataset further highlights this trend, with AGIIN-MAF achieving an impressive 91.2% mAP50 under occlusion, compared to YOLO11n’s 70.5% and RT-DETR-L’s 73.2%, despite all models starting near 98-99% without occlusion. This consistent outperformance across diverse datasets illustrates AGIIN-MAF’s robust adaptability and effectiveness in detecting occluded pests, making it a promising solution for real-world agricultural applications. To provide a more intuitive understanding of these performance dynamics, the visual comparisons presented in **Figures 4**–**6** map the mAP50 scores across the respective datasets. These bar charts distinctly illustrate that while all evaluated models perform competitively under optimal, non-occluded conditions, the baseline architectures suffer a precipitous decline in accuracy when confronted with environmental occlusions. In stark contrast, the bars representing our AGIIN-MAF model demonstrate a significantly shallower performance drop, effectively bridging the accuracy gap caused by structural noise and proving the efficacy of the integrated feature restoration mechanism across diverse agricultural scenarios. Considering computational efficiency alongside performance, **Table 1** and **Table 2** provide insights into the trade-offs between model complexity and practical deployment. YOLO11n stands out as the lightest model, with 2.62M parameters and 6.6 GFLOPs in both training and testing, yet its poor occlusion handling (e.g., mAP50 of 64.1% on the custom dataset and 39.4% on IP102) limits its utility. Conversely, RT-DETR-L, with 33M parameters and GFLOPs of 108.4 (training) and 103.9 (testing), offers better occlusion performance (71.3% and 33.1% mAP50, respectively) but at a high computational cost, rendering it less feasible for real-time use. AGIIN-MAF strikes an optimal balance, with 5.86M parameters and a testing GFLOPs of 6.6 (matching YOLO11n), despite a higher training GFLOPs of 108.6. This efficiency, combined with its high mAP50 under occlusion (80.2% on the custom dataset and 51.2% on IP102), positions AGIIN-MAF as a practical and robust choice for pest detection tasks, effectively bridging the gap between lightweight design and strong performance in challenging conditions.

**Figure 7:**
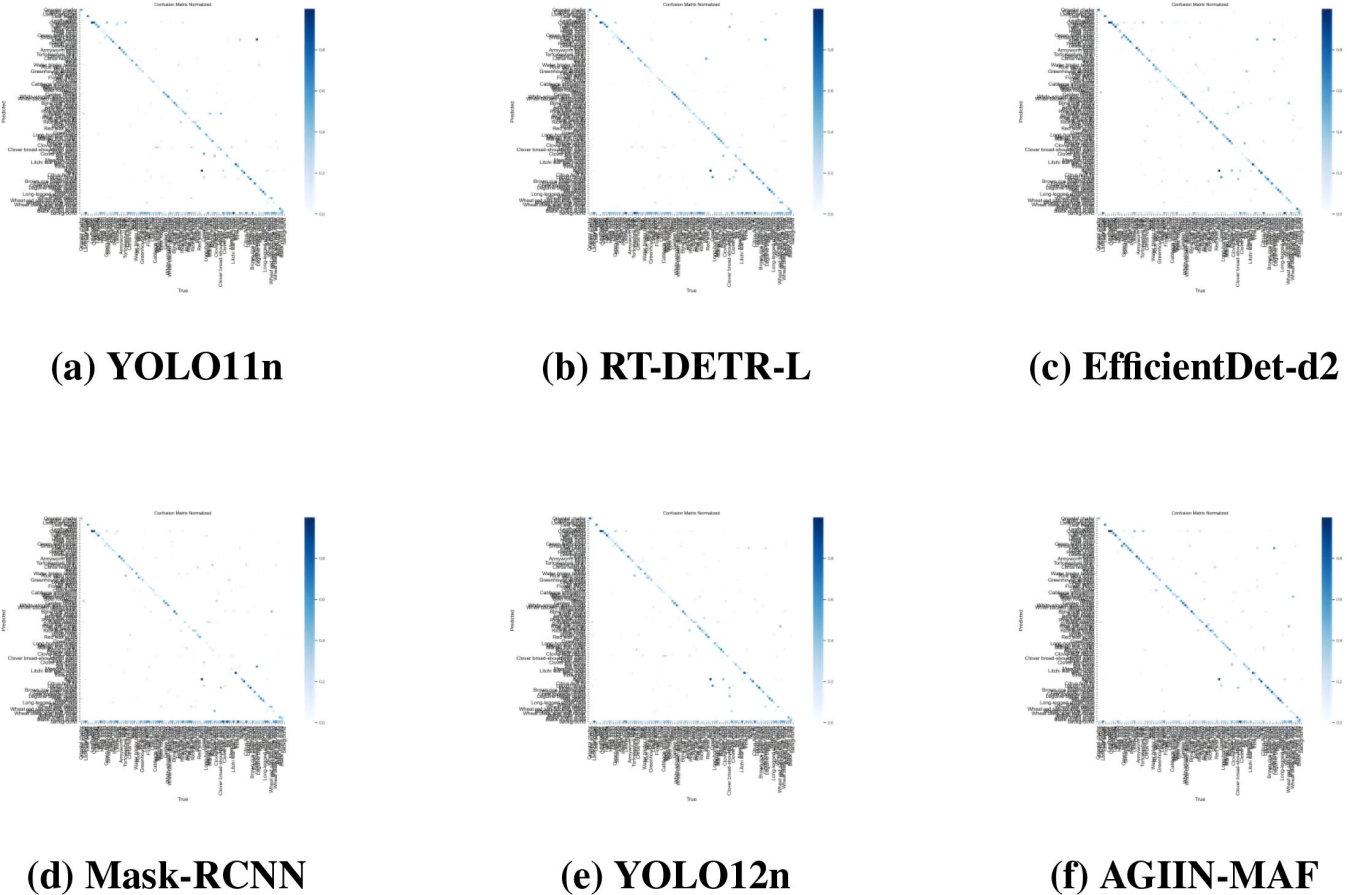
Confusion matrix plots of the six evaluated methods on the IP102 dataset under random occlusion. The vertical axis represents the True Class (Ground Truth), and the horizontal axis represents the Predicted Class. The normalized values on the diagonal indicate the classification accuracy for each pest category, where a darker blue color signifies higher precision in distinguishing specific species under challenging conditions.

In the confusion matrix plots presented in **Figure 7**, we observe significant differences in the classification performance of the six models under occlusion conditions on the IP102 dataset. The diagonal of each confusion matrix represents correct classifications, with darker blue indicating higher accuracy, while off-diagonal elements signify misclassifications. Among the models, AGIIN-MAF (**Figure 7f**) stands out with the deepest and most consistent blue along its diagonal, reflecting its superior mAP50 of 51.2%. This indicates that AGIIN-MAF maintains a high level of classification accuracy even when pests are partially occluded, with minimal misclassifications across various pest categories. In contrast, YOLO11n (**Figure 7a**) exhibits a moderate blue intensity on the diagonal but shows noticeable misclassifications, particularly between similar pest categories, which aligns with its lower mAP50 of 39.4%. RT-DETRL (**Figure 7a**) performs the worst, with a lighter and less consistent diagonal and a more scattered distribution of misclassifications, corresponding to its mAP50 of 33.1%. This suggests that RT-DETRL struggles significantly under occlusion, leading to frequent confusion between different pest types. EfficientDet-d2 (**Figure 7c**) and Mask-RCNN (**Figure 7d**) display intermediate performance, with their diagonals showing a mix of moderate to light blue, indicating varying degrees of classification accuracy and misclassification patterns. YOLO12n (**Figure 7e**), while an improvement over YOLO11n, still exhibits some misclassifications, though less pronounced than in RT-DETR-L. Overall, the confusion matrices clearly demonstrate that AGIIN-MAF’s architecture, likely due to its integration of advanced feature extraction and attention mechanisms, enables it to outperform the other models in maintaining classification accuracy under challenging occlusion conditions.

**Figure 8:**
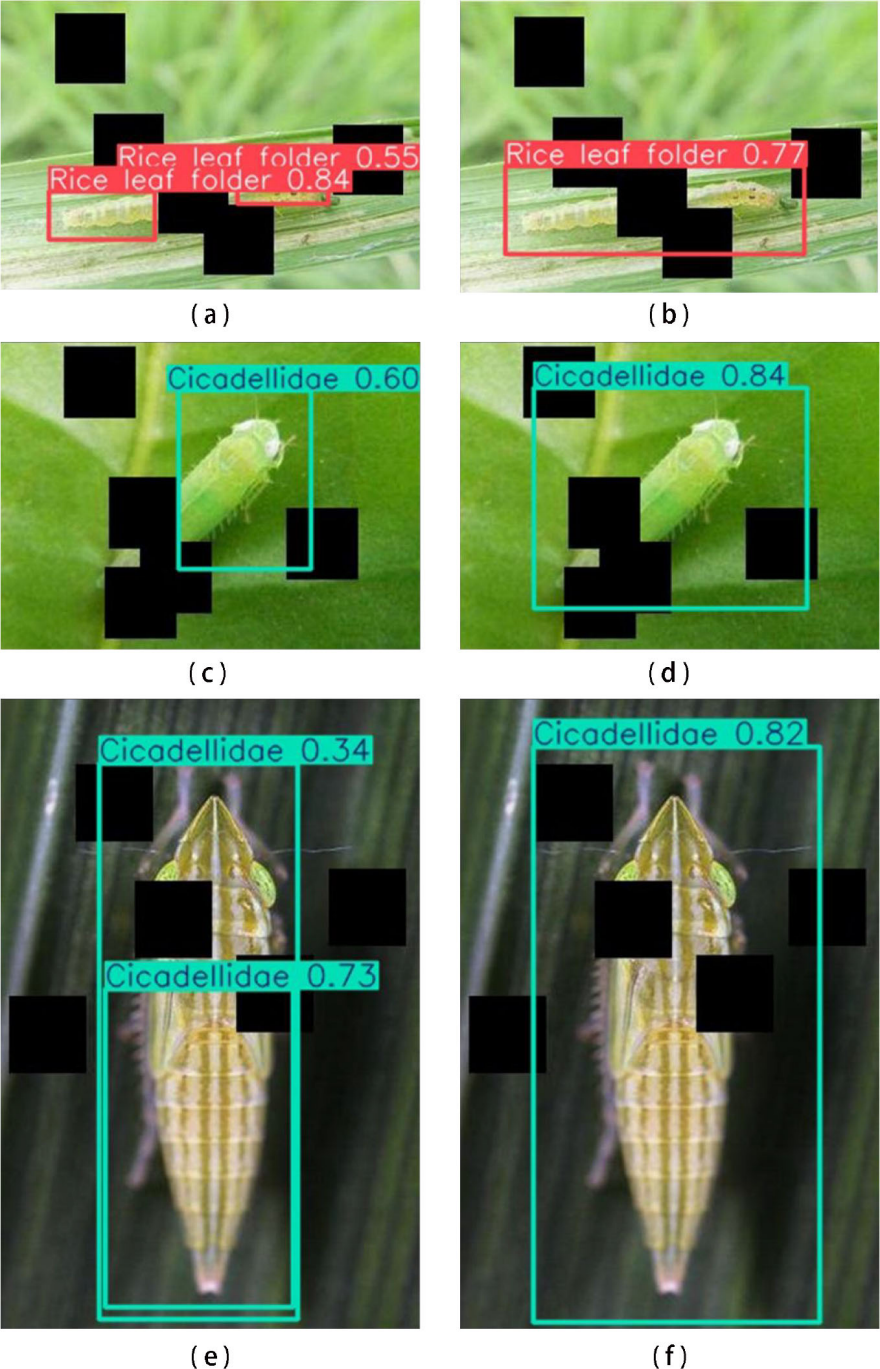
Comparison of detection effects between YOLO11n and AGIIN-MAF under occlusion. The left side represents YOLO11n, while the right side represents AGIIN-MAF.

Additionally, as can be seen from **Figure 8**, when detecting occluded pests, the YOLO11n model on the left has some noticeable shortcomings. Firstly, it sometimes incorrectly detects a single actual target as two separate targets. This may be due to occlusion causing certain features of the target to be split apart, making it difficult for the model to correctly integrate these features into a complete object. Secondly, the YOLO11 model sometimes fails to detect occluded targets completely, i.e., it only detects a part of the target while ignoring other occluded parts. This may be due to the model’s insufficient learning of features from occluded areas, preventing it from accurately identifying and locating all parts of the target. Moreover, the YOLO11 model may also incorrectly detect half of a target as a whole, which may be due to the model’s inadequate understanding of the overall features of the target, preventing it from correctly judging the boundaries and integrity of the target.

The AGIIN-MAF model effectively mitigates occlusion challenges, demonstrating superior performance in detecting partially hidden pests. Firstly, the AGIIN-MAF method, by introducing CAE and multi-branch feature fusion, enables the model to more accurately identify and locate all parts of the target. Even when the target is occluded, it can effectively integrate dispersed features into a complete object, thus avoiding the situation of incorrectly detecting a single target as two targets. Secondly, this method enhances the model’s ability to learn features from occluded areas, allowing the model to more comprehensively detect all parts of the target. Even if some parts are occluded, it can accurately identify and locate them, thus avoiding incomplete target detection. Furthermore, the AGIIN-MAF method optimizes the model’s understanding of the overall features of the target, enabling it to more accurately judge the boundaries and integrity of the target, thus avoiding the situation of incorrectly detecting half of a target as a whole. Overall, the AGIIN-MAF method demonstrates higher accuracy and robustness in handling occluded pest detection tasks, effectively coping with various complex occlusion scenarios and providing strong support for the accuracy and reliability of pest detection tasks in practical applications.

In **Table 6**, we selected YOLO11n as the baseline model. The symbol × indicates that the component is not included, while ✓ indicates that the component is included. Specifically, When AGIIN is removed, the two branches are simply fused by element-wise addition followed by a 1×1 convolution (with a fixed weight of 0.5 applied to the auxiliary features before adding). When the Auxiliary Model is not included, the randomly occluded images are either left untreated or processed only through the CAE (Convolutional Autoencoder) to serve as the features of the auxiliary branch. All results were tested on the test set containing random occlusions.

**Table 6:** Results of ablation experiment (/%).

CAE	AGIIN	Auxiliary model	Precision	Recall	mAP50	mAP50-95
×	×	×	74.2	55.9	64.1	32.5
✓	×	×	76.9	62.4	71.3	38.6
×	✓	×	72.3	58.4	68.7	35.9
×	×	✓	68.7	61.9	66.4	34.1
✓	✓	×	79.2	65.7	77.9	48.3
✓	×	✓	75.7	64.3	74.5	45.8
×	✓	✓	76.8	67.2	72.6	42.5
✓	✓	✓	81.8	72.4	80.2	50.1

By analyzing the data in **Table 6**, we can observe that each of these three components has improved the accuracy of the original model in detecting occluded pests to varying degrees. Adding CAE, AGIIN, and the Auxiliary Model individually improved mAP50 to 71.3%, 68.7%, and 66.4%, respectively—outperforming the baseline model. Particularly, the addition of CAE alone shows the most significant improvement, indicating that even without AGIIN and the Auxiliary Model, CAE enables the main model to learn a certain ability to detect occluded objects. The CAE’s encoding-decoding architecture enables efficient feature extraction and restoration of occluded information.

When two components are added respectively, CAE and AGIIN together jump to 77.9%, which is noticeably more than just adding their individual gains. This tells us that plain addition lets a lot of remaining noise from badly occluded regions leak into the main model, whereas the adaptive gate in AGIIN knows when to trust the reconstruction and when to block the garbage. Once we finally bring back the full auxiliary branch (so the model has to live with heavily corrupted inputs throughout training), we get another solid bump to 80.2%. In the end the gap to clean-image performance shrinks to only about 4%, which is pretty remarkable for such severe random occlusion.

**Figure 9:**
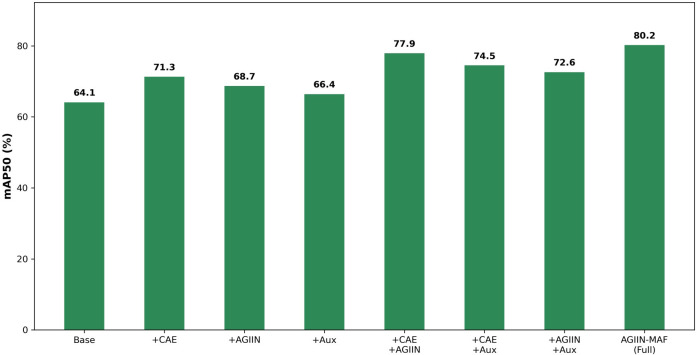
Visualization of the step-by-step mAP50 improvements in the ablation experiment.

All in all, the three pieces are genuinely complementary: CAE does the heavy lifting of repair, AGIIN acts as a smart filter that prevents the repair from backfiring, and the complete auxiliary branch forces the main model to truly get used to broken images rather than just peeking at cleaned-up versions every now and then. The combined application of CAE, AGIIN, and the Auxiliary Model significantly improves the model’s performance in detecting occluded pests. The Auxiliary Model, through the processing of randomly occluded images, provides additional training samples for the model, enhancing its adaptability to occlusion scenarios. At the same time, the Auxiliary Model can also supervise and guide the Main Model, further optimizing the model’s detection performance. This multi-component collaborative approach not only improves the model’s detection accuracy for occluded objects but also enhances its robustness and generalization ability in complex scenarios, providing a strong guarantee for the accuracy and reliability of pest detection tasks in practical applications. The incremental performance gains analyzed during the ablation study are intuitively depicted in **Figure 9**, which tracks the evolution of the model from the base architecture to the fully integrated AGIIN-MAF framework. This step-by-step visualization clarifies the specific contribution of each module, visually confirming that the most significant jump in detection accuracy occurs when the Convolutional Autoencoder and Adaptive Gate Information Integration Network are simultaneously employed to jointly handle feature inpainting and adaptive information flow.

### 3.3 Testing phase

To evaluate the model’s adaptability during testing, we constructed a dataset comprising images under various environmental conditions, including fog, rain, snow, bright, dark, Gaussian noise, salt-and-pepper noise, motion blur, and JPEG corruption, with 673 images per condition, amounting to a total of 6,057 images. TENT (Wang et al., 2021b), CoTTA (Wang et al., 2022), and RoTTA (Yuan et al., 2023) were originally designed for image classification tasks. In this work, we apply their core ideas to the YOLO11n baseline and make only minimal necessary adjustments to accommodate the object detection task.

**Figure 10:**
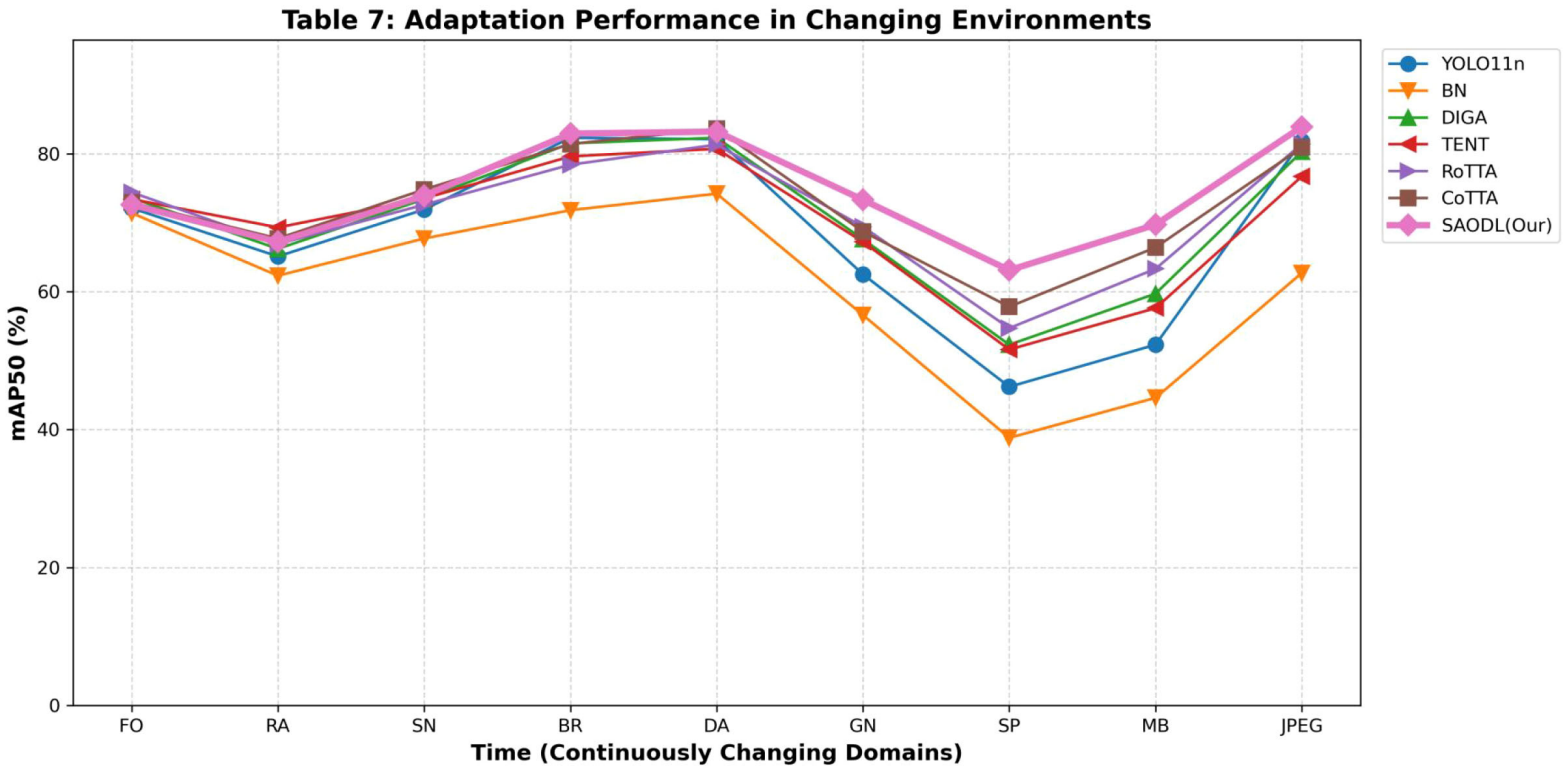
Long-term adaptation curves of different methods across continuously changing environments.

**Figure 11:**
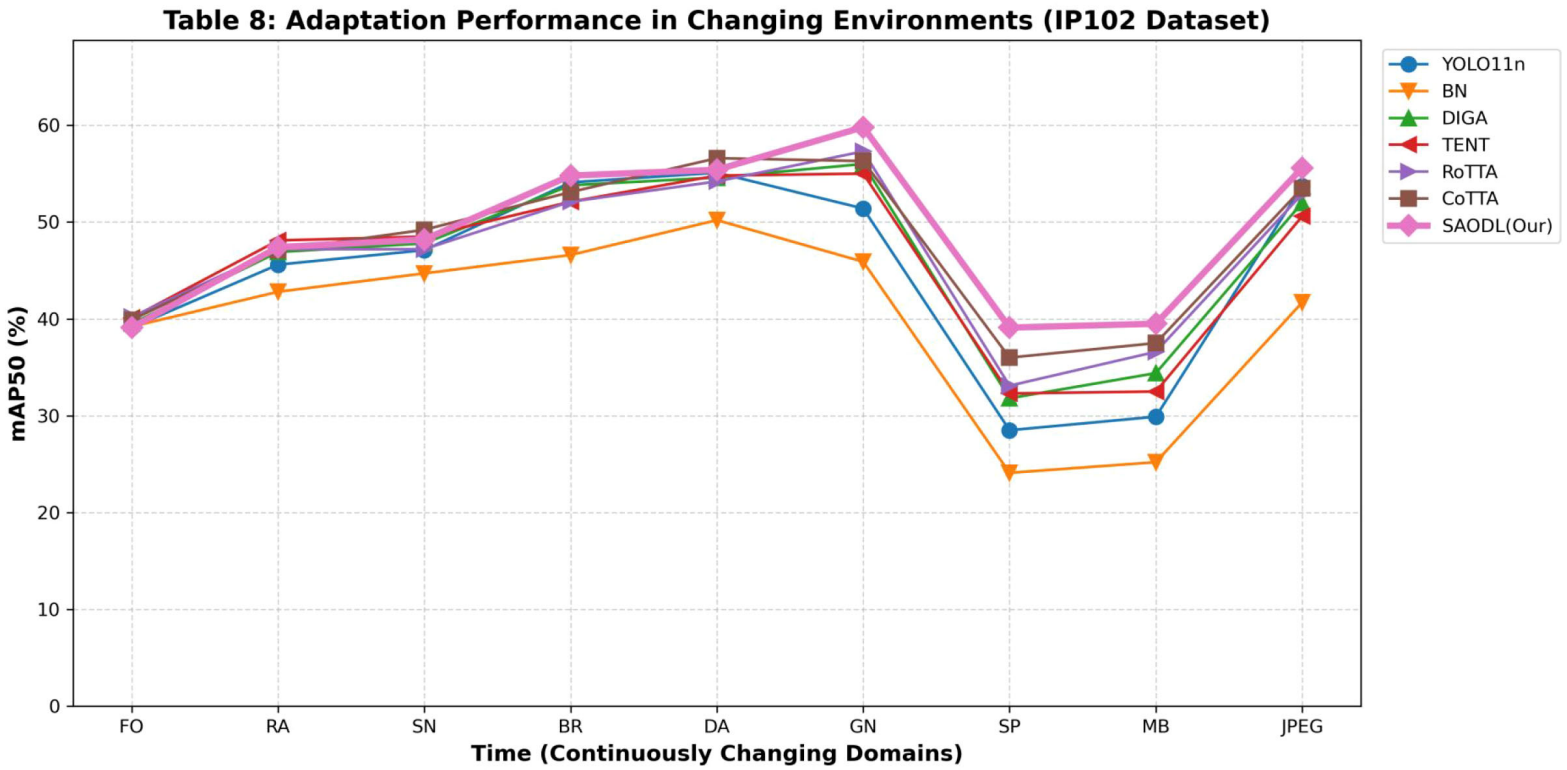
Adaptation performance trends on the IP102 dataset across continuously changing environments.

In **Table 7**, we conducted a detailed comparison of the SAODL method with other TTA methods. The highest values in each column are marked in red font, while the bold font represents the second-highest values in each column. Looking at the overall results, SAODL achieved remarkable performance with the highest average mAP50 of 74.4%. In contrast, the BN method, due to its limited adaptability, performed poorly with an average mAP50 of only 61.1%, even below the baseline level. The DIGA method, with its high efficiency and unique ability to avoid error accumulation, showed excellent performance in the early stages and remained relatively stable without significant accuracy drops due to error accumulation in later stages. The TENT method can quickly adapt to new data distributions, but since it was originally designed for classification tasks, its overall performance is relatively poor and it fails to fully realize its potential. The RoTTA and CoTTA methods performed relatively well, with the CoTTA method standing out due to its strong continuous adaptation ability, achieving an average mAP50 of 72.7%. Although the RoTTA method also performed well overall, it was mainly designed for long-term scenarios and performed slightly worse than CoTTA in rapidly changing environments, with an average mAP50 of 71.4%. Moreover, in the constantly changing environment, when transitioning from Gaussian noise to salt and pepper noise, the performance of all models declined to varying degrees, but SAODL was the least affected during this phase, which fully demonstrates the effectiveness and superiority of the parameter selection update strategy in SAODL. Looking at the overall trend, SAODL was slightly behind other methods in the early stages, mainly because we adopted a selective parameter update strategy, updating only a portion of domain-sensitive parameters, which led to a relatively slower adaptation speed in new environments compared to other models. However, as the model gradually adapted and adjusted in multiple different environments, SAODL eventually stabilized in the first position. This result strongly confirms that our SAODL method successfully retained domain-invariant parameters, ensuring that the model would not experience significant accuracy fluctuations in different environments, achieving good stability and adaptability. The dynamic adaptability of our method is comprehensively reflected in the long-term performance curves shown in **Figure 10**. Across a temporal sequence of varying environmental stressors, such as fog, snow, and digital noise, the SAODL strategy consistently maintains the highest and most stable mAP50 levels. Unlike traditional test-time adaptation methods that often exhibit erratic fluctuations or suffer from catastrophic forgetting over extended periods, our selective parameter optimization approach ensures a stable balance between preserving source knowledge and adapting to new domains. In the analysis of the **Table 8**, we compared the SAODL method with other Test-Time Adaptation methods using the IP102 dataset. The highest values in each column are highlighted in red, while the second-highest values are marked in bold. Overall, SAODL achieved the highest average mAP50 of 48.8%, demonstrating outstanding performance across diverse environmental conditions. In contrast, the BN method, limited by its poor adaptability, recorded a mere 40.0% average mAP50, falling below the baseline YOLO11n’s 44.9%. DIGA maintained stable performance in specific columns like BR and GN, with an average mAP50 of 46.3%, though it lacked consistency across all conditions. TENT, originally designed for classification tasks, excelled in RA with the highest value and SN with the second-highest, but its overall adaptability was constrained, resulting in an average mAP50 of 46.0%. RoTTA performed strongly in FO with the highest value and GN with the second-highest, achieving an average mAP50 of 46.7%, yet it slightly underperformed in rapidly changing environments. CoTTA, a strong contender, secured the highest values in SN and DA and the second-highest in SP, MB, and Avg, with an average mAP50 of 47.7%. During the transition from Gaussian noise to salt and pepper noise, all models experienced performance drops, but SAODL was the least affected, achieving the highest mAP50 in SP at 39.1% and GN at 59.8%, underscoring the effectiveness of its parameter selection update strategy. Early on, SAODL lagged slightly in stages like FO and RA due to its selective parameter update approach, which prioritizes long-term stability over rapid adaptation. However, as it adjusted to multiple environments, SAODL’s performance surged, ultimately taking the lead. This trend highlights how SAODL, by preserving domain-invariant parameters, minimizes accuracy fluctuations, delivering exceptional stability and adaptability in continuously changing environments, making it an ideal choice for real-world applications. To further validate the reliability of the SAODL strategy under severe distribution shifts, **Figure 11** visualizes the adaptation process on the challenging IP102 dataset. The line graph distinctly illustrates that while all models struggle with the inherent difficulty of the IP102 distribution, the SAODL algorithm effectively curbs severe performance degradation. The stable upward trajectory of our method confirms its efficacy in continuous agricultural monitoring, where the model must autonomously adapt to unpredictable weather and lighting variations without revisiting the source data.

**Table 7:** The performance (mAP50/%) of various methods in the continuously changing environment.

Time	−−−−−−−−−−−−−−−−−−−−−−−−−−−−−−−−−−−−−−−−−−−−−−−−−−−−−−→									
	FO	RA	SN	BR	DA	GN	SP	MB	JPEG	Avg
YOLO11n	72.1	65.1	71.9	82.3	82.1	62.5	46.2	52.3	81.8	68.5
BN	71.4	62.3	67.7	71.8	74.2	56.6	38.8	44.6	62.7	61.1
DIGA	73.6	66.2	73.4	81.5	82.3	67.6	52.3	59.7	80.3	70.8
TENT	73.4	69.3	73.5	79.6	80.7	67.2	51.6	57.6	76.7	69.9
RoTTA	74.4	66.9	72.6	78.4	81.3	69.4	54.7	63.3	81.4	71.4
CoTTA	72.9	67.7	74.8	81.4	83.6	68.7	57.8	66.4	80.9	72.7
SAODL(Our)	72.6	67.2	73.9	82.9	83.2	73.3	63.1	69.7	83.9	74.4

**Table 8:** The performance (mAP50/%) of various methods in the continuously changing environment, using the IP102 dataset.

Time	−−−−−−−−−−−−−−−−−−−−−−−−−−−−−−−−−−−−−−−−−−−−−−−−−−−−−−→									
	FO	RA	SN	BR	DA	GN	SP	MB	JPEG	Avg
YOLO11n	39.1	45.6	47.1	54.1	55.1	51.4	28.5	29.9	53.7	44.9
BN	39.2	42.8	44.7	46.6	50.2	45.9	24.1	25.2	41.7	40.0
DIGA	39.5	46.9	47.8	53.8	54.6	56.0	31.8	34.4	52.0	46.3
TENT	40.1	48.1	48.5	52.1	54.8	55.0	32.3	32.5	50.6	46.0
RoTTA	40.2	47.2	47.2	52.1	54.2	57.3	33.1	36.6	52.9	46.7
CoTTA	39.9	47.1	49.2	53.1	56.6	56.3	36.0	37.5	53.5	47.7
SAODL(Our)	39.1	47.4	48.2	54.8	55.4	59.8	39.1	39.5	55.6	48.8

**Figure 12:**
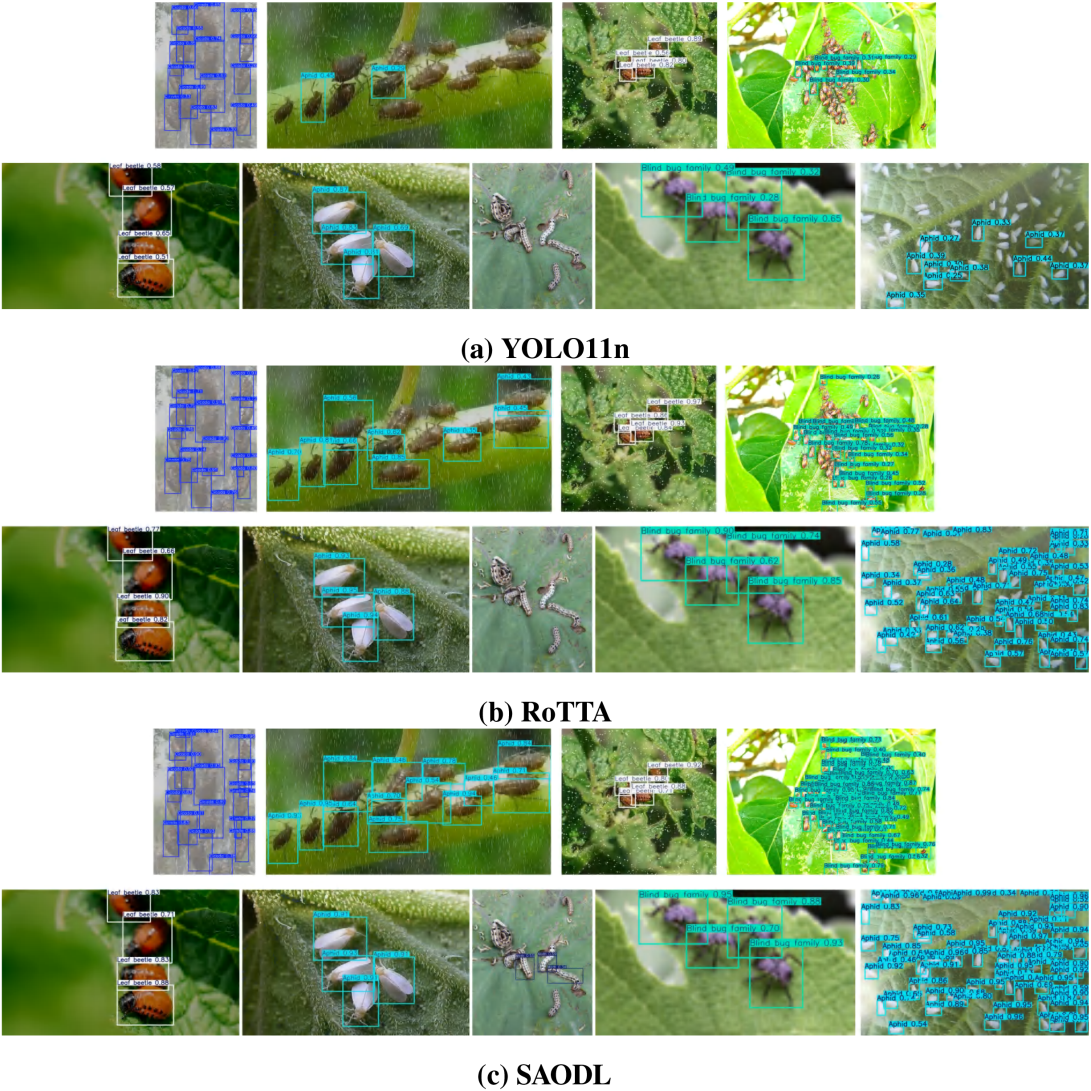
Detection results of YOLO11n, RoTTA, and SAODL across nine distinct environmental challenges: fog (col 1), rain (col 2), digital noise (col 3), overexposure (col 4), motion blur (col 5), low light (col 6), complex backgrounds (col 7), partial occlusion (col 8), and dense occlusion (col 9).

After carefully examining **Figure 12**, it becomes clear that the YOLO11 baseline model, when faced with complex cross-domain changes, although capable of identifying some target objects, generally has a low detection confidence. Moreover, the model exhibits a significant number of missed detections, which to some extent limits its effectiveness and reliability in practical applications. In stark contrast, the RoTTA method demonstrates superior performance under the same testing conditions. It not only increases the confidence of object detection but also successfully identifies more target objects, thereby achieving significant progress in the comprehensiveness and accuracy of object detection. However, even so, the RoTTA method is not without flaws. Especially when facing environments with special interference characteristics such as salt and pepper noise, the detection capability of this method drops noticeably, leading to a large number of target objects not being effectively detected, a deficiency that could have serious consequences in practical applications.

In response to the aforementioned issues, our proposed SAODL method has made breakthroughs in several key aspects. Firstly, in terms of confidence, the SAODL method has seen a significant improvement compared to both the YOLO11 baseline model and the RoTTA method. This enhancement means that the SAODL method is more confident and reliable in determining the existence of target objects, thereby providing a more solid foundation for subsequent decision-making and processing. Secondly, in multiobject environments, the SAODL method has essentially resolved the issue of missed detections. This indicates that the method can more comprehensively identify all target objects in the scene, regardless of their size, shape, or position, and can accurately detect them. This improvement in capability is of vital importance for enhancing the overall performance and practicality of the system.

However, the SAODL method is not infallible in all circumstances. Particularly in environments with salt and pepper noise, although the method can detect the target objects in the image, the precision of the detection boxes is not entirely satisfactory. This may be due to the characteristics of the salt and pepper noise environment. Salt and pepper noise disrupts image content by adding random white and black pixels, making precise localization of objects challenging. In such cases, even if the detection algorithm can identify the approximate location of the target objects, it is difficult to accurately determine the boundaries of the target objects due to the noise, leading to deviations in the detection boxes.

Despite the aforementioned challenges, overall, the SAODL method has achieved significant success in addressing the issue of pest detection in constantly changing cross-domain environments. It not only improves detection confidence and accuracy but also shows strong robustness in dealing with complex environmental interference. These advantages make the SAODL method have broad application prospects in practical applications, providing strong technical support for research and practice in related fields.

## 4 Discussion

The AGIIN-MAF method proposed in this study effectively enhances the detection of occluded pests by leveraging the strengths of both the main and auxiliary models. The integration of CAE and AGIIN in the auxiliary model plays a crucial role in improving the robustness of the detection system. The results from the training phase demonstrate a significant improvement in the detection accuracy of occluded objects, highlighting the effectiveness of our approach in handling real-world scenarios where pests may be partially hidden. It should be noted that while random occlusion is employed as a data augmentation strategy during training to force the model to learn robust feature reconstruction, the evaluation is not limited to synthetic noise. The datasets used in this study (e.g., IP102 and Forestry Pest) naturally encompass the structural and semantic complexities of real-world environments, such as leaf interposition and insect clustering. The robust performance of AGIIN-MAF shown in 8 and 12, particularly in detecting overlapping and partially obscured pests, indicates that the model’s ability to recover information from random masks successfully generalizes to complex, non-random structural occlusions encountered in actual agricultural production.Furthermore, the cross-dataset generalization capability of our framework is theoretically supported by the SAODL adaptation mechanism. By utilizing the Fisher Information Matrix (FIM) to decouple domain-invariant knowledge from domain-sensitive features, the model can specifically identify and update the weights that are sensitive to background and lighting shifts while freezing the core detection representations. This selective adaptation explains why the model maintains high accuracy when transitioning between datasets with distinct characteristics (e.g., from the specialized Forestry Pest dataset to the broad IP102 dataset), effectively bridging the gap created by significant distributional differences in environment and scale.

In the testing phase, the SAODL strategy proves to be highly effective in adapting the model to new environments. By selectively updating parameters based on their sensitivity to domain changes, our method maintains the model’s generalization ability while enhancing its adaptability. This is particularly important in agricultural settings where environmental conditions can vary widely, and the model needs to perform reliably across different scenarios.

Compared to other TTA methods, SAODL shows superior performance in terms of average mAP50, indicating its effectiveness in balancing stability and adaptability. The method’s ability to retain knowledge from the source domain while adapting to the target domain is a significant advantage, as it prevents catastrophic forgetting and ensures consistent performance.

## 5 Conclusion

This study presents a comprehensive approach to improving pest detection in agriculture, focusing on the challenges of occluded pests and varying environmental conditions. The AGIIN-MAF method, with its innovative use of a convolutional autoencoder and an adaptive gate information integration network, significantly enhances the model’s ability to detect pests even when they are partially occluded. The introduction of the SAODL strategy during the testing phase further strengthens the model’s adaptability to new environments, ensuring robust performance across different conditions.

The results of our experiments confirm the effectiveness of the proposed methods, with the AGIIN-MAF achieving an mAP50 of 80.2% in detecting occluded objects and the SAODL strategy achieving an average mAP50 of 74.4% across various environmental conditions. These findings underscore the potential of the proposed approach to advance effective and reliable pest management solutions in agricultural applications.

Future research can continue to advance along several interrelated dimensions, focusing on both methodological refinement and practical deployment. On the methodological side, further enhancing the representational capacity of AGIIN-MAF through deeper cross-scale semantic modeling and more expressive feature interaction mechanisms may strengthen the model’s ability to capture fine-grained textures, complex occlusion patterns, and subtle visual cues under diverse field conditions. Likewise, the SAODL strategy could be expanded by incorporating more precise estimations of domain shifts and stronger self-supervised constraints, enabling the model to maintain stable performance when confronted with previously unseen environmental variations. In terms of application, extending the proposed framework to a wider variety of crops, pest species, and seasonal conditions, as well as validating its robustness across different ecological and geographical regions, will be essential for demonstrating its broader generalizability. Furthermore, integrating the system with UAV-based monitoring platforms, edge-computing devices, and agricultural IoT infrastructures may facilitate the development of a real-time, data-driven closed-loop optimization paradigm, allowing the model to evolve continuously through online updates and feedback. Such advancements hold promise for pushing pest detection technologies toward large-scale, intelligent, and autonomously adaptive agricultural management systems.

## Data Availability

The datasets presented in this study can be found in online repositories. The names of the repository/repositories and accession number(s) can be found in the article/supplementary material.
